# Medial Prefrontal Cortex Glutamate Is Reduced in Schizophrenia and Moderated by Measurement Quality: A Meta-analysis of Proton Magnetic Resonance Spectroscopy Studies

**DOI:** 10.1016/j.biopsych.2021.06.008

**Published:** 2021-06-12

**Authors:** Jason Smucny, Cameron S. Carter, Richard J. Maddock

**Affiliations:** Department of Psychiatry and Behavioral Sciences, University of California Davis, Davis, California.

## Abstract

**BACKGROUND:**

Magnetic resonance spectroscopy studies measuring brain glutamate separately from glutamine are helping elucidate schizophrenia pathophysiology. An expanded literature and improved methodologies motivate an updated meta-analysis examining effects of measurement quality and other moderating factors in characterizing abnormal glutamate levels in schizophrenia.

**METHODS:**

Searching previous meta-analyses and the MEDLINE database identified 83 proton magnetic resonance spectroscopy datasets published through March 25, 2020. Three quality metrics were extracted—Cramér–Rao lower bound (CRLB), line width, and coefficient of variation. Pooled effect sizes (Hedges’ *g*) were calculated with random-effects, inverse variance-weighted models. Moderator analyses were conducted using quality metrics, field strength, echo time, medication, age, and stage of illness.

**RESULTS:**

Across 36 datasets (2086 participants), medial prefrontal cortex glutamate was significantly reduced in patients (*g* = −0.19, confidence interval [CI] = −0.07 to −0.32). CRLB and coefficient of variation quality subgroups significantly moderated this effect. Glutamate was significantly more reduced in studies with lower CRLB or coefficient of variation (*g* = −0.44, CI = −0.29 to −0.60, and *g* = −0.43, CI = −0.29 to −0.57, respectively). Studies using echo time ≤20 ms also showed significantly greater reduction in glutamate (*g* = −0.41, CI = −0.26 to −0.55). Across 11 hippocampal datasets, group differences and moderator effects were nonsignificant. Group effects in thalamus and dorsolateral prefrontal cortex were also nonsignificant.

**CONCLUSIONS:**

High-quality measurements reveal consistently reduced medial prefrontal cortex glutamate in schizophrenia. Stricter CRLB criteria and reduced nuisance variance may increase the sensitivity of future studies examining additional regions and the pathophysiological significance of abnormal glutamate levels in schizophrenia.

Evidence from genetic, molecular, ultrastructural, physiological, animal modeling, pharmacological, and cognitive studies implicates abnormalities of glutamatergic neurotransmission in schizophrenia (1-8). For some glutamatergic processes, the evidence suggests elevated activity in schizophrenia. Reduced activity, however, is suggested for others. A recent reanalysis of postmortem studies found that abnormalities involving components of glutamatergic neurotransmission in the prefrontal cortex (PFC) vary in their direction of change at different levels of anatomical resolution (3).

Proton magnetic resonance spectroscopy (MRS) studies measuring regional brain tissue glutamate content, especially recent studies using improved MRS methods, can help elucidate the pathophysiology of schizophrenia (9-13). There is not yet a consistent pattern of findings of abnormal tissue glutamate content in specific brain regions in schizophrenia. In a 2013 meta-analysis, Marsman *et al*. (14) reported a modest but significant reduction in medial PFC (mPFC) glutamate across 9 studies in schizophrenia patients. A subsequent meta-analysis of 14 studies of mPFC glutamate by Merritt *et al*. (15) did not replicate the earlier report, but noted a nonsignificant reduction in glutamate in patients with schizophrenia. Neither study found significant differences in glutamate in other brain regions, although some differences were seen across studies reporting a composite measure combining glutamate and glutamine levels (glx). Sydnor and Roalf (16) recently reported a meta-analysis confined to studies using ultrahigh field MR systems (≥7T). In 59 datasets across all brain regions, they observed significantly reduced glutamate (but not glx) in schizophrenia. The same subjects, however, were often represented in multiple brain regions. Region-specific effects, furthermore, were not reported. In a meta-analysis of 13 studies targeting the dorsolateral PFC, Kaminski *et al*. (17) found no overall difference between schizophrenia patients and control subjects. Their meta-analysis, however, treated glutamate and glx measurements as equivalent. Glx is often reported when the investigators judge that measurement quality is insufficient for quantifying glutamate separately from glutamine. Alternatively, glx may be reported when the investigators are interested in the combined pool of glutamate and glutamine. Glutamate and glutamine, however, may each be affected differently in schizophrenia. Notably, the Merritt *et al*. (15) meta-analysis covered studies published before April 1, 2015. Many additional studies of brain glutamate in schizophrenia have appeared since that time, most of which have used improved MRS methodology. It is possible that high-quality measurements of brain glutamate separately from glutamine will reveal a consistent pattern of regionally abnormal glutamate levels in schizophrenia.

Many factors influence the quality of glutamate measurements in MRS studies, including scanning parameters, post-processing methods, and subject motion. Several metrics are available that reflect the final quality of the overall spectra and of the glutamate measurements in particular. These include line width, range and variability of the final glutamate values (summarized by calculating the coefficient of variation [COV]), and Cramér–Rao lower bound (CRLB) for glutamate. The latter estimates the precision of the model fitting procedure for glutamate as the lower limit of the variance of glutamate estimates derived from fitting the basis set to the metabolite spectrum (18). We reasoned that among the larger set of MRS studies in schizophrenia now available, we could identify a subset of studies that achieved relatively high-quality measurements of glutamate separately from glutamine. The primary goals of this meta-analysis were to 1) characterize any abnormalities in regional brain glutamate levels in schizophrenia and 2) test the hypothesis that abnormal glutamate is most evident in studies in which glutamate measurement quality surpasses an empirically identifiable threshold. We also examined whether field strength, echo time, medication status, age, or phase of illness moderate effect size in schizophrenia.

## METHODS AND MATERIALS

### Study Selection

Previous meta-analyses (14-17) were searched for published, English-language, single-voxel, proton MRS studies that reported glutamate values from any brain region in healthy volunteers and patients with schizophrenia or a schizophrenia spectrum illness. The Merritt *et al*. (15) meta-analysis included studies up to April 1, 2015. For subsequent studies, the MEDLINE database was searched to identify journal articles published between April 1, 2015, and March 25, 2020, using the following search terms: *MRS* or *magnetic resonance spectroscopy* and 1) *schizophrenia* or 2) *psychosis* or 3) *schizoaffective*, while excluding reviews. This search yielded 1076 records for screening and 200 full-text articles for eligibility assessment (Figure S1).

### Meta-analysis

For each brain region studied, author JS extracted and author RJM verified data. Extracted data included sample sizes, means and SDs of glutamate values, means and SDs of glutamate CRLB, and means and SDs of line width (quantified as full width at half maximum [FWHM] of singlet peaks). We also extracted field strength, echo time (TE), mean duration of illness, mean patient age, and medication status. When studies reported separately on multiple patient and control groups, they were treated as independent datasets. When multiple patient groups were compared with a single control group, the patient groups were combined and treated as a single dataset. For longitudinal studies, only the values given for the first time point were included. For studies reporting partially overlapping samples, only data from the study with the largest sample were included. Studies not reporting glutamate were excluded (102 studies, 65 reporting glx only). When glutamate values normalized to both water and creatine were reported, the normalization method producing the lowest COV for glutamate averaged across groups was used. COV of creatine-normalized glutamate was lower in all 7 cases. One study reporting raw glutamate values without any normalization was excluded. When bilateral glutamate values were reported, only the hemisphere most commonly studied for that region was included (right for hippocampus and left for all other regions). When studies reported on 2 voxels in the same region, the voxel with higher glutamate COV was excluded. Two studies measuring glutamate during cognitive tasks were excluded. Only studies generating one-dimensional spectra at a single TE were included (2 excluded).

Effect size for each dataset was calculated as Hedges’ *g*, which corrects for small sample sizes (19). The meta-analysis used an inverse variance-weighted, random-effects model to calculate the pooled effect size. For determining significance, τ^2^ was calculated by the restricted maximum likelihood method. The analysis was conducted with JASP software (20), which uses the R-based metafor package as its computational engine. Heterogeneity across studies was quantified as *I*^2^, and a χ^2^ test of the *Q* statistic tested for significant departure from homogeneity. Meta-analytic hypothesis testing was performed only in brain regions for which ≥7 datasets were available.

We hypothesized that any true difference in glutamate values in schizophrenia would be most evident in studies with relatively better quality glutamate measurements. For CRLB and FWHM, we calculated the mean + 2 SD for the patient and control groups and then averaged those values. Approximately 95% of subjects would have values below this level for each study. If only the mean was reported, SD was imputed using the median of the SD/mean ratios from all other studies reporting both mean and SD. For the COV of glutamate values, we calculated the average COV (as SD/mean) of the patient and control groups. We reasoned that the relationship between measurement quality and effect size would be logistic (sigmoid), rather than linear. That is, we expected that there would be a quality threshold beyond which pooled effect sizes would become larger and more consistent. Formally, we hypothesized that there was a quality threshold *Q* for which the meta-analytic result would be significantly stronger in studies surpassing *Q* than for those falling short of *Q*. To identify the quality threshold, we plotted the inverse variance-weighted pooled effect sizes from a moving sample (*k* = 7) running from the lowest- to the highest-quality studies for each quality metric (analogous to a moving average). A best-fitting, four-parameter, logistic function was fit to these pooled effect sizes (21), and the resulting equation was used to generate a logistic transform of the quality measure. The inflection point in the logistic function defined the quality threshold *Q* and was used to stratify studies into low- and high-quality subgroups for each metric (Supplemental Methods). Including these subgroups as moderating variables in the overall meta-analysis tested our hypothesis that the meta-analytic result would be stronger in studies with higher quality measurements. Because three different quality metrics were examined, we used a Bonferroni-corrected α of 0.05/3 = 0.017 for testing this hypothesis. Secondary meta-analyses were conducted on the individual subgroups. Because this procedure requires a minimum of approximately 14 studies (twice the number in the moving sample of 7 studies) to reliably identify higher- and lower-quality subgroups, it was only performed for brain regions with ≥14 datasets available.

Field strength and echo time (as log TE) were examined as potential moderators using metaregression. Clinical variables, including mean patient age, recent-onset psychosis (defined as mean duration of illness ≤24 months) versus chronic illness, and medication status were also examined. In examining whether these factors account for heterogeneity across datasets, moderator analysis was limited to brain regions for which ≥10 datasets were available, as recommended by the Cochrane Handbook (22). For all significant results, small study bias was tested using the Egger test, and the multivariate outlier detection battery (23,24) was used to screen for outliers.

## RESULTS

After excluding 143 studies (Table S1), 86 datasets from 57 studies reporting brain glutamate were included in the final sample. Of these, 36 reported on the mPFC (including the anterior cingulate cortex) (12,13,25-53), and 11 reported on the hippocampus (27,29,47,54-60). Regions totaling <10 datasets included the thalamus (9 datasets), dorsolateral PFC (8 datasets), striatum (5 datasets), frontal white matter (4 datasets), occipital cortex (4 datasets), and other regions with ≤2 datasets (Table S2). Meta-analytic hypothesis testing was performed only for datasets from mPFC, hippocampus, thalamus, and dorsolateral PFC (all ≥7 datasets). Exploratory meta-analyses were performed for other regions.

### Medial PFC

#### Overall Findings.

**Figure 1.**
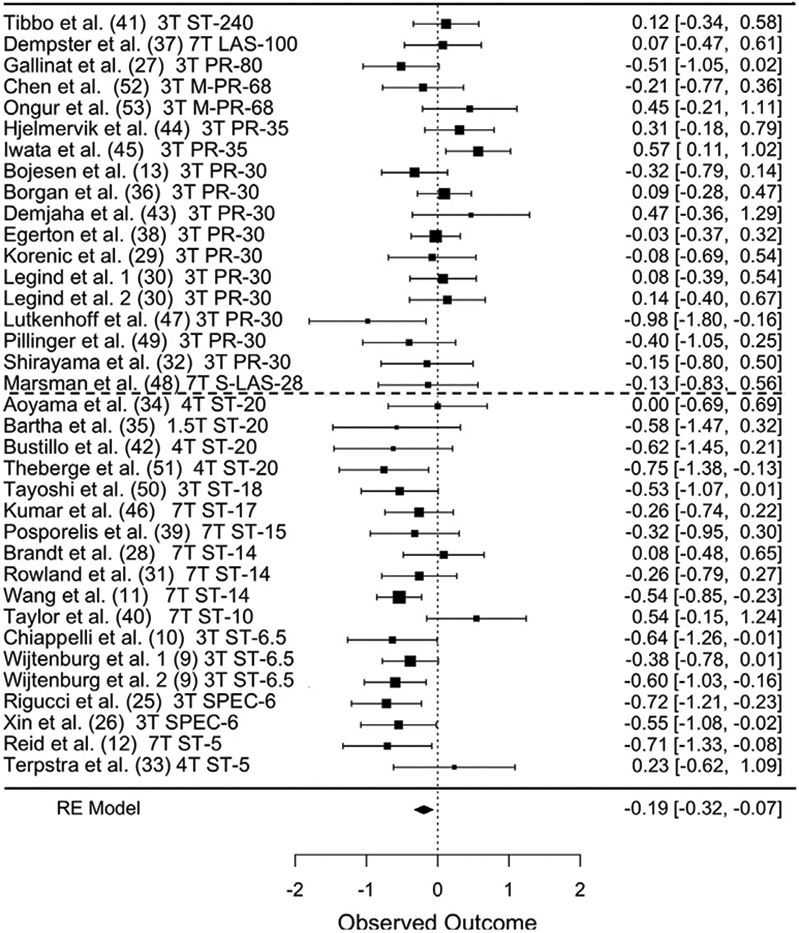
Forest plot of 36 datasets reporting medial prefrontal cortex glutamate, ordered from longest to shortest echo times. Publication, field strength, sequence, and echo time are listed at left. Hedges’ *g* and [95% confidence interval] are at center and right. Dashed line indicates echo time median split. LAS, LASER; M-PR, MEGA-PRESS; PR, PRESS; RE, random effects; SPEC, SPECIAL; ST, STEAM.

Across 36 datasets (1022 patients, 1064 controls), we found a small but significant reduction in glutamate in schizophrenia (*g* = −0.19; 95% confidence interval (CI), −0.07 to −0.32; *Q*_1_ = 9.1, *p* = .003; heterogeneity: *I*^2^ = 48%, *p* < .001) (Figure 1 and Table 1; Table S3).

#### Effects of Glutamate Measurement Quality Metrics.

**Figure 2.**
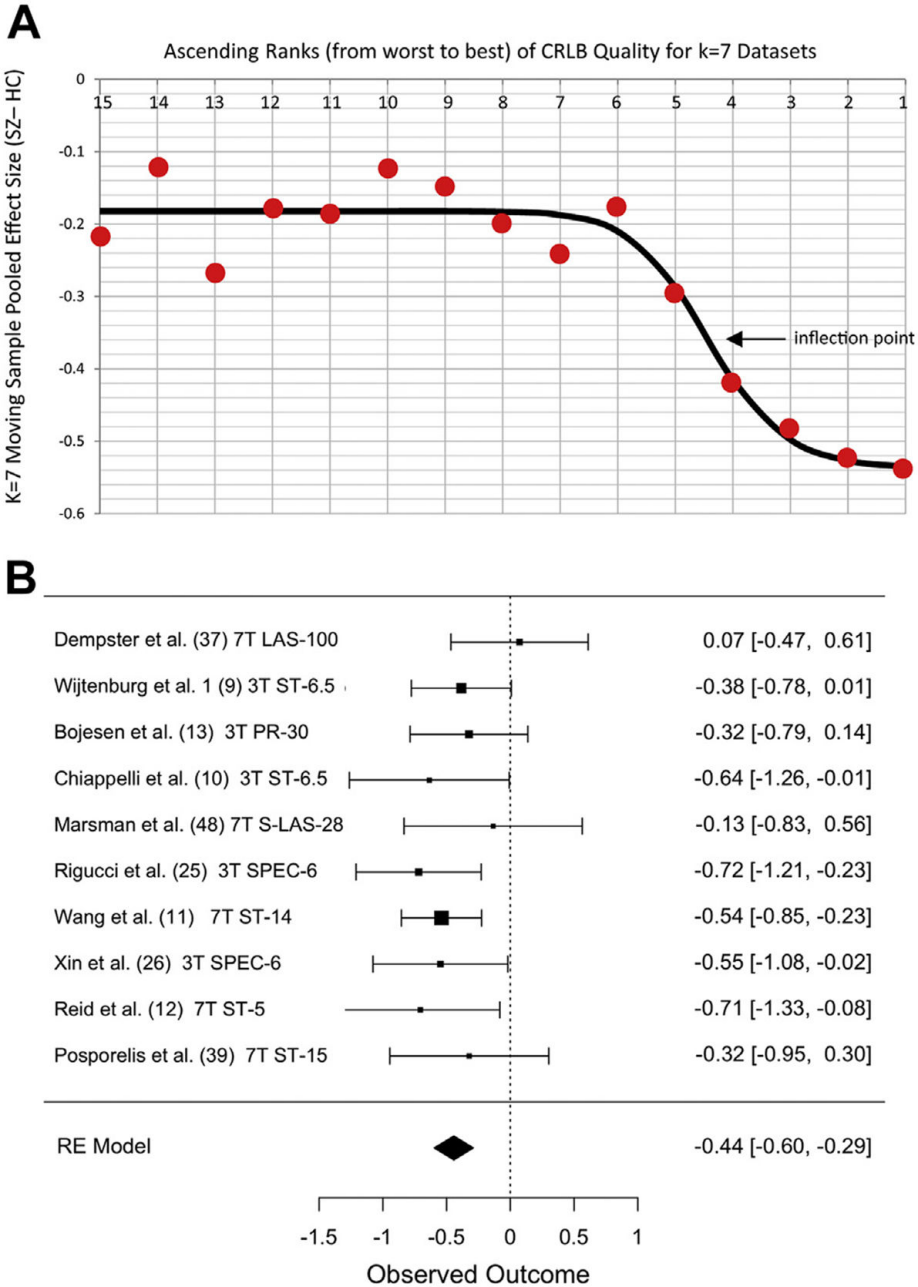
Stronger evidence for reduced glutamate in schizophrenia in datasets with lower Cramér–Rao lower bound (CRLB). **(A)** Red circles represent the moving sample pooled effect size (Hedges’ *g*) from 7 datasets in ascending ranks of CRLB from poorer (left) to better (right) along the x-axis. Circle #15 is the effect size for 7 datasets ranked 15–21. Circle #1 is for studies ranked 1–7 (total *k* = 21). Black line is best-fitting logistic function. Inflection point is quality threshold separating low- and high-quality datasets. **(B)** Forest plot of 10 low-CRLB (high-quality) datasets showing pooled effect size *g* = −0.44. Datasets ordered by CRLB (best at bottom). Other notes as in Figure 1. HC, healthy control; RE, random effects; SZ, schizophrenia.

**Figure 3.**
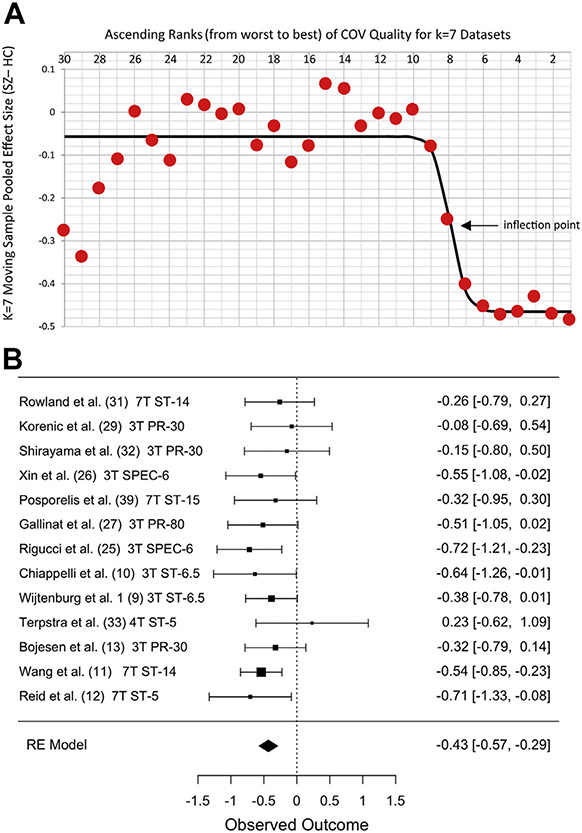
Stronger evidence for reduced glutamate in schizophrenia in datasets with lower coefficients of variation (COVs). **(A)** Red circles represent the moving sample pooled effect size from 7 datasets in ascending ranks of COV from poorer (left) to better COV (right) along the x-axis. Circle #30 is the effect size for seven datasets ranked 30–36. Circle #1 is for datasets ranked 1–7 (total *k* = 36). Black line is best-fitting logistic function. Inflection point is threshold separating low- and high-quality datasets. **(B)** Forest plot of 13 low-COV (high-quality) datasets showing pooled effect size *g* = −0.43. Datasets ordered by COV (best at bottom). Other notes as in Figure 1. HC, healthy control; RE, random effects; SZ, schizophrenia.

Among glutamate measurement quality metrics, mean CRLB + 2 SD was available for 21 datasets (6 with SD imputed), mean FWHM + 2 SD for 19 datasets (no imputations), and mean COV for all 36 datasets. Datasets were dichotomized into higher- versus lower-quality subgroups for each metric, as described in Methods and Materials. For all three metrics, the best-fitting logistic transform fit the data well, with *r*^2^ values ranging from 0.79 to 0.93 (Figures 2A and 3A). This procedure identified 10 high-quality datasets for CRLB (mean + 2 SD ≤ 7%), seven high-quality datasets for FWHM (mean + 2 SD ≤ 0.058 ppm), and 13 high-quality datasets for COV (mean COV ≤ 10%). Datasets not reporting CRLB or FWHM were included in the low-quality subgroup for those metrics (15 datasets for CRLB, 17 for FWHM).

Moderator analyses showed that effect sizes differed significantly between low- and high-quality subgroups for CRLB and COV (for CRLB: omnibus model *Q*_1_ = 7.7, *p* = .006; heterogeneity: *I*^2^ = 35%, *p* = .018; and for COV: omnibus model *Q*_1_ = 10.1, *p* < .001; heterogeneity: *I*^2^ = 29%, *p* = .033). For both quality metrics, mPFC glutamate was significantly more reduced in datasets with better measurement quality. Secondary analyses of the individual subgroups showed that glutamate was significantly reduced only in the subgroups with higher-quality measurements (Table 1 and Figures 2B and 3B). Subgroups based on FWHM quality showed a similar nonsignificant trend at our corrected α (omnibus model *Q*_1_ = 4.9, *p* = .027; heterogeneity: *I*^2^ = 41%, *p* = .007).

We also conducted exploratory analyses in which datasets not reporting CRLB or FWHM were excluded rather than assigned to the low-quality subgroups. For FWHM, the effect size was significantly greater in 7 high-quality compared with 12 lower-quality datasets (omnibus model *Q*_1_ = 6.2, *p* = .013; heterogeneity: *I*^2^ = 42%, *p* = .028), and glutamate was significantly reduced only in the high-quality subgroup (Table 1). CRLB showed a similar nonsignificant trend at our corrected α (omnibus model *Q*_1_ = 4.14, *p* = .042; heterogeneity: *I*^2^ = 22%, *p* = .13).

#### Effects of Field Strength and Echo Time.

Moderator analysis using field strength and log TE as metaregressors showed no effect of field strength but a significant effect of TE (omnibus model *Q*_1_ = 9.4, *p* = .002; heterogeneity: *I*^2^ = 34%, *p* < .03). mPFC glutamate was more strongly reduced in schizophrenia in datasets using shorter echo times. We further explored this effect via a secondary analysis with a median split into subgroups of 18 datasets with TE ≤20 ms (range 5–20) and 18 datasets with TE ≥28 ms (range 28–240). A significant reduction in mPFC glutamate in schizophrenia was observed only in the shorter TE subgroup (Table 1). A horizontal dashed line in Figure 1 shows the median split point for TE. An exploratory analysis showed that normalization method (water vs. creatine) did not significantly moderate effect size (*Q*_1_ = 0.6, *p* = .67).

#### Effects of Medication Status, Age, and Phase of Illness.

In 28 of 36 datasets, patient medication status could be categorized as 100% unmedicated (*k* = 5) or ≥80% medicated (*k* = 23). Eight datasets did not fit these categories and were excluded from this analysis. Medication status did not significantly moderate effect size (omnibus model *Q*_1_ = 0.31, *p* = .58; heterogeneity: *I*^2^ = 48%, *p* < .003), although mPFC glutamate was significantly reduced across the medicated datasets (*g* = −0.27) but not across the smaller sample of unmedicated datasets (*g* = −0.15). Mean patient age was not significant as a metaregressor (omnibus model *Q*_1_ = 0.02, nonsignificant; heterogeneity: *I*^2^ = 49%, *p* = .001). Mean duration of illness was #24 months in 9 studies (recent onset) and >24 months in 27 studies. Phase of illness was not a significant moderator of effect size (omnibus model *Q*_1_ = 0.62, *p* = .43; heterogeneity: *I*^2^ = 48%, *p* < .001). mPFC glutamate was significantly reduced in both recent-onset (*g* = −0.28, CI, −0.09 to −0.48; *Q*_1_ = 8.1, *p* = .004; heterogeneity: *I*^2^ = 24%, *p* < .32) and chronic (*g* = −0.16, CI, −0.01 to −0.32; *Q*_1_ = 4.1, *p* = .04; heterogeneity: *I*^2^ = 53%, *p* < .001) patient subgroups.

### Hippocampus

Across 11 hippocampal datasets (173 patients, 259 controls), no significant difference between patients and controls was observed (Table 1; Table S4 and Figure S2). Metaregression analysis showed no effect of either field strength or log TE. The distributions of these regressors, however, were very limited. There were no studies above 3T, and 8 of the 11 studies used TE between 30 and 35 ms. Three datasets were categorized as ≥80% unmedicated and 6 datasets as 100% medicated (2 datasets excluded). Medication status did not significantly moderate effect size (Table S4). Similarly, 4 datasets were categorized as recent onset and 7 as chronic. Neither phase of illness nor mean patient age significantly moderated effect size (Table S4).

### Other Regions

No significant group differences were observed across 9 datasets from the thalamus or across 8 datasets from the dorsolateral PFC (Table 1; Figures S3 and S4). Exploratory moderator analyses found no effects in these regions. Exploratory meta-analyses of glutamate in brain regions with *k* = 4 and *k* = 5 datasets (striatum, frontal white matter, and occipital cortex) found no significant group effects for these regions (Figures S5-S7).

### Small Study Bias and Influential Outliers

Funnel plots showed no evidence of small study bias influencing any of the significant effects reported above or in Table 1 (all *p* > .15, Egger’s test). Similarly, influential outliers were observed only in the mPFC metaregression with log TE. Removing these outliers increased the significance of the metaregression.

## DISCUSSION

In this largest meta-analysis yet, to our knowledge, of brain glutamate measured separately from glutamine using proton MRS in a psychiatric disorder, we observed a small but highly significant reduction in mPFC glutamate in schizophrenia. We found no consistent difference in glutamate in the hippocampus, thalamus, or dorsolateral PFC. Only the mPFC had sufficient datasets for a formal test of our hypothesis that glutamate abnormalities are most evident in studies where measurement quality surpasses an empirically identifiable threshold. This hypothesis was confirmed. Reduced mPFC glutamate in schizophrenia was statistically significant at the meta-analytic level only across studies where glutamate CRLB or mean COV met empirical quality thresholds (95% of CRLB values ≤7%, and mean COV ≤ 10%, respectively). In such studies, Hedges’ *g* pooled effect size was ~ −0.43, with minimal heterogeneity (*I*^2^ = 0%). Across studies not meeting these thresholds, heterogeneity was substantial, and no significant difference in glutamate was observed (*g* ~ −0.08). The weighted mean percent difference in mPFC glutamate between patients and controls was ~5% across studies with high-quality measurements, compared to ~2% across all studies.

Significant reduction in mPFC glutamate in schizophrenia aligns with converging evidence from multiple modalities implicating prominent involvement of the region in the pathophysiology of the disorder. In addition to glutamate, previous MRS meta-analyses, for example, have found reduced levels of other metabolites in the mPFC in schizophrenia, including *N*-acetylaspartate, myo-inositol, and glutathione, with *N*-acetylaspartate (widely considered a marker of neuronal integrity) having the largest effect size (61-64). Structural studies have found regionally broad reductions in gray matter volume in several mPFC subregions, including the dorsal and ventral paralimbic and limbic anterior cingulate cortices (65). Furthermore, these deficits appear to be significant across all phases of illness, including the at-risk state (65). Previous work also indicates that the mPFC is functionally aberrant in schizophrenia, including electroen-cephalographic evidence suggesting reduced mPFC error-related negativity (66), and functional magnetic resonance imaging evidence suggesting reduced activation during various executive function and emotion perception tasks (67,68). A meta-analysis of mPFC activation after cognitive remediation in schizophrenia across working memory, emotion regulation, reality monitoring, and verbal fluency tasks also observed increased activation of the region after treatment, suggesting that its function may be normalized by intervention (69).

Abnormalities affecting glutamatergic neurotransmission are embedded in a web of homeostatic processes (4). While abnormalities implicating both increased and decreased cortical glutamatergic activity have been observed in schizophrenia (3-6), the current meta-analytic finding suggests that processes leading to reduced tissue glutamate predominate in the mPFC. Studies aimed at identifying clinical, cognitive, and electrophysiological correlates of individual differences in mPFC glutamate in schizophrenia may help delineate the pathophysiological significance of this abnormality. The current findings show that measurement quality may be a critical factor in such studies. Although most MRS studies examined used a CRLB inclusion threshold of <20%, moderator analysis showed that studies with lower range of CRLB values (≤7%) were significantly more sensitive to reduced mPFC glutamate than studies reporting a higher range of CRLB values or not reporting CRLB values. If a similar pattern is observed in other brain regions as more studies become available, then a more conservative approach to CRLB thresholds may be worth considering in studies of glutamate abnormalities in schizophrenia. It is worth noting that although high CRLB values reliably indicate measurement problems, low CRLB values do not necessarily indicate high-quality measurements. For example, shortcomings in the overall model used for spectral fitting can produce low CRLB values but poorly estimated metabolites. Glutamate COV values ≤10% also distinguished studies sensitive to reduced mPFC glutamate in schizophrenia. The degree of variation reported in some studies was patently implausible (threefold to fourfold variation across healthy volunteers), suggesting inadequate quality-based exclusion criteria. When studies not reporting FWHM were excluded from the measurement quality analysis, FWHM values ≤0.058 ppm also reliably distinguished studies sensitive to reduced mPFC glutamate in schizophrenia. In future MRS studies of schizophrenia, optimized scanning conditions, careful voxel shimming, management of subject motion, and unbiased exclusion of distorted spectra and outlier values could improve FWHM values and minimize nuisance variance (70-72).

Our finding that studies using TEs ≤20 ms were significantly more sensitive to reduced mPFC glutamate in schizophrenia confirms an earlier report (14). Although very short TE spectra contain more potentially overlapping signal from macromolecules and lipids, they may provide more accurate glutamate measurements by reducing losses from T2 relaxation and minimizing the J-evolution of glutamate’s coupled resonances (73). For each of the three quality metrics examined here (CRLB, FWHM, and COV), mPFC studies identified as higher quality used pulse sequences with significantly shorter echo times (quantified as log TE) than those identified as lower quality. This suggests that the larger effect size with very short TE sequences is due, in part, to better measurement quality. One intriguing possibility is that very short TE sequences are more sensitive to glutamate in fast-relaxing microenvironments, such as synaptic vesicles (74,75). If so, lower glutamate seen only at very short TEs could reflect reduced vesicular glutamate in schizophrenia (76). The evidence supporting microenvironment-based differences in MRS-visibility of glutamate, however, comes from early MRS studies (74,75,77), and definitive studies of this model using current MRS methods have not yet appeared. Further research is needed to understand why reduced mPFC glutamate in schizophrenia is most reliably observed in studies using TE ≤20 ms. Nonetheless, it is worth noting that TE differences are confounded with localization sequence differences. The very short TE studies all used STEAM (or SPECIAL) sequences, while all but one of the longer TE studies used PRESS (or LASER) sequences, precluding separation of the effects of TE from those of localization sequence.

The meta-analytic finding of reduced mPFC glutamate does not exclude heterogeneity in glutamatergic pathophysiology among individual patients. Clinical factors associated with such heterogeneity may include age, stage or severity of illness, and antipsychotic medication use. An association between antipsychotic medication and glutamate levels has been suggested by subject-level data from previous studies (38,78). A new mega-analysis by Merritt *et al*. (79) published late in our review process observed a significant negative correlation between medial frontal cortex glutamate level and antipsychotic dose, with lower glutamate levels associated with higher antipsychotic doses across all medicated patients. This contrasts with the current meta-analytic finding that the medication status of patient samples did not significantly moderate effect sizes for glutamate. Several factors may account for this apparent inconsistency. The analysis of Merritt *et al*. (79) excluded unmedicated patients, reporting only on dose-related effects in patients taking antipsychotics. In the current analysis, effect sizes from studies of unmedicated patients were directly compared with effect sizes from studies of medicated patients. In addition, the samples of medicated patients included in Merritt *et al*. (79) and those in the current study were largely nonoverlapping, with only 16% of the latter sample overlapping with the former. Importantly, the number of unmedicated patient datasets in the current meta-analysis was small (5), and only 1 met the high-quality threshold for any quality metric. These factors may have diminished our sensitivity for detecting a significant difference between effect sizes owing to medicated versus unmedicated status. Future studies of unmedicated patients may clarify whether reduced mPFC glutamate is present to a similar degree in unmedicated and medicated patients with schizophrenia.

Neither mean patient age within a dataset nor stage of illness was found to modify glutamate effect sizes. Considerable evidence suggests that brain glutamate level declines with age (13,80). Only the moderating effect of age on patient versus control differences, however, can be examined at a meta-analytic level. An earlier meta-analysis of mPFC glutamate reported that patient versus control effect sizes were significantly more negative in studies of older patients (14). The current meta-analysis did not confirm this finding, either across all 36 studies or when confined to short TE or high-quality measurement subgroups. Similarly, the recent mega-analysis by Merritt *et al*. (79) found that medial frontal cortex glutamate declined with age at similar rates in schizophrenia patients and control subjects.

Illness severity is another potential source of heterogeneity in mPFC glutamate. The current analysis could not address this question with meta-analytic data. Merritt *et al*. (79), however, observed some clinical associations with medial frontal cortex glutamate, showing that it was positively associated with symptom severity and negatively associated with global functioning in schizophrenia patients. These findings suggest that mPFC glutamate may be associated with a pathogenic process in the illness and that reduced glutamate may reflect an adaptive or regulatory response to this process.

A major limitation of this study is that abnormal glutamate and the role of measurement quality in demonstrating it could only be shown in the mPFC. The negative result for hippocampal studies may reflect the paucity of datasets with high-quality metrics or very short TEs. The hippocampus poses a particular challenge for acquiring high-quality glutamate measurements (71). The small number of datasets in other brain regions similarly limited our ability to characterize other possible glutamate abnormalities. As new glutamate studies of hippocampal and other regions are published, reliable abnormalities may become evident in additional brain regions in schizophrenia.

In conclusion, meta-analysis of 36 studies showed significantly reduced mPFC glutamate in schizophrenia. Empirically derived quality thresholds using either CRLB or COV significantly moderated this effect, as did TE. Significantly reduced mPFC glutamate was evident at the meta-analytic level only across studies with high measurement quality or very short TE. Careful attention to these factors will be necessary in future MRS studies to explicate the pathophysiological significance of abnormal brain glutamate levels in schizophrenia.

## Supplementary Material

Supplementary material

## Figures and Tables

**Table 1.** Meta-analytic Results for Brain Regions and Quality Metric Subgroups

Region	Subgroup	Datasets	Cases	Healthy Control Subjects	Effect Size (95% CI)	*p* Value	Heterogeneity *I*^2^, *p* Value
mPFC	All datasets	36	1022	1064	−0.19 (−0.07 to −0.32)	.003	48%, <.001
	CRLB^a^ ≤ 7%	10	321	350	−0.44 (−0.29 to −0.60)	<.001	0%, .58
	CRLB^a^ > 7%	11	309	336	−0.18 (0.04 to −0.40)	.10	45%, .046
	CRLB not stated	15	392	378	−0.02 (0.18 to −0.22)	.85	45%, .032
	COV^b^ ≤ 10%	13	388	410	−0.43 (−0.29 to −0.57)	<.001	0%, .76
	COV^b^ > 10%	23	634	654	−0.07 (0.10 to −0.23)	.42	48%, .006
	FWHM^c^ ≤ 0.058	7	202	229	−0.48 (−0.28 to −0.67)	<.001	0%, .83
	FWHM^c^ > 0.058	12	474	505	−0.08 (0.13 to −0.28)	.47	59%, .005
	FWHM not stated	17	346	330	−0.18 (0.01 to −0.38)	.07	39%, .052
	TE ≤ 20 ms	18	487	516	−0.41 (−0.26 to −0.55)	<.001	18%, .24
	TE ≥ 28 ms	18	535	548	0.0 (0.15 to −0.15)	.99	26%, .10
Hippocampus	All datasets	11	173	259	0.17 (0.61 to −0.27)	.44	78%, .001
Thalamus	All datasets	9	281	318	0.09 (0.29 to −0.11)	.39	29%, .27
DLPFC	All datasets	8	245	310	−0.06 (0.30 to −0.41)	.76	72%, .003

COV, coefficient of variation (of measured glutamate values); CRLB, Cramér–Rao lower bound for fitting glutamate resonances to their basis set; DLPFC, dorsolateral prefrontal cortex; FWHM, full width at half maximum; mPFC, medial PFC; TE, echo time.

a Mean + 2 SDs of CRLB values, averaged across patients and control subjects.

b Average of COV values from patient and control groups.

c Mean + 2 SDs of FWHM values in parts per million, averaged across patients and control subjects.
