# Supplementary material for: Medial Prefrontal Cortex Glutamate Is Reduced in Schizophrenia and Moderated by Measurement Quality: A Meta-analysis of Proton Magnetic Resonance Spectroscopy Studies
Source: Biol Psychiatry. Author manuscript; Available in PMC 2022 Nov 1. (PMC9303057; doi:10.1016/j.biopsych.2021.06.008)
Supplement: Supplementary material [file NIHMS1823474-supplement-Supplementary_material.pdf]

Medial Prefrontal Cortex Glutamate Is Reduced in Schizophrenia and Moderated by Measurement Quality: A Meta-analysis of Proton Magnetic Resonance Spectroscopy Studies

Supplement

Contents of the Supplement

Supplemental Methods: Empirical method for identifying quality thresholds

Supplemental Results: Exploratory analysis of signal-to-noise as a quality metric

Table S1: Excluded studies and reasons for exclusion

Table S2: Included studies of regions for which < 10 datasets are available

Table S3: Medial Prefrontal Glutamate - Study Characteristics, Glutamate Data, and Quality Metrics

Table S4: Hippocampal Glutamate - Study Characteristics, Glutamate Data, Quality Metrics, and Moderator Effects

Table S5: Thalamic Glutamate - Study Characteristics, Glutamate Data, and Quality Metrics

Table S6: Dorsolateral Prefrontal Glutamate - Study Characteristics, Glutamate Data, and Quality Metrics

Figure S1: PRISMA Flow Chart

Figure S2: Forest plot of meta-analysis of 11 hippocampal studies

Figure S3: Forest plot of meta-analysis of 9 thalamus studies

Figure S4: Forest plot of meta-analysis of 8 dorsolateral prefrontal cortex studies

Figure S5: Forest plot of exploratory meta-analysis of 5 striatal studies

Figure S6: Forest plot of exploratory meta-analysis of 4 frontal white matter studies

Figure S7: Forest plot of exploratory meta-analysis of 4 occipital cortex studies

Supplemental References: References cited in Supplement

Supplemental Methods – Empirical method for identifying quality thresholds.

We examined three metrics sensitive to the quality of the glutamate measurements for each study: mean + 2 SD for Cramer-Rao lower bound (CRLB), mean + 2 SD for singlet line width (FWHM), and mean COV for glutamate. For each metric, we averaged the values for the patient and control groups. We hypothesized that glutamate measurement quality would have a moderating effect on the meta-analytic results across studies comparing schizophrenia patients to healthy volunteers. Formally, we hypothesized there was a quality threshold Q, for which the meta-analytic result would be significantly stronger in studies surpassing Q than for those falling short of Q. To identify the quality threshold Q in an unbiased manner, we first ranked the studies for each metric. We then calculated the inverse variance-weighted pooled effect sizes from a moving sample of studies (k = 7) running from the lowest to the highest quality studies for each quality metric (analogous to a moving average). A best-fitting, 4-parameter, logistic function was fit to this series of pooled effect sizes using the computational resource at https://mycurvefit.com/ using the following equation:

Y = d + ((a – d)/(1+(X/c)^b))

Where Y = the pooled effect size (k=7) and X = the rank of the set of seven adjacent studies for the quality metric being examined. The best fitting four parameters (a, b, c, and d) for each of the quality metrics is shown below.

	a	b	c	d	Inflection point (Q)
CRLB	-0.1822	26.31	17.57	-0.5382	-.3602
FWHM	+0.0343	4.518	18.60	-0.8647	-.4152
COV	-0.0568	67.00	29.09	-0.4653	-.2610

Parameter “a” is the asymptote of the pooled effect size for the lowest quality datasets, and parameter “d” is the asymptote of the pooled effect size for the highest quality datasets for each metric. These best fitting parameters were used to generate a logistic transform of the ranks of each quality metric. The empirical quality threshold Q was identified as the inflection point in the logistic transform curve. The inflection point (Q) is the midpoint between parameters “a” and “d” (thus Q = (a + d)/2). This point Q was used to stratify studies into low and high quality subgroups for each metric. All studies included in a set of 7 ranked studies for which the moving pooled effect size (k = 7) was more negative than Q were stratified into the high quality subgroup for that quality metric. All other studies were stratified into the lower quality subgroup.

Supplemental Results – Exploratory analysis of signal-to-noise as a quality metric.

Spectral signal-to-noise (SNR) was not included as an *a priori* quality metric for testing the hypothesis that the meta-analytic result would be significantly stronger in studies surpassing an empirically identified threshold for measurement quality. We chose this approach in order to limit multiple comparisons for testing this hypothesis. It was our opinion, *a priori*, that SNR would be the least discriminating of the four quality metrics commonly reported (COV, CRLB, FWHM, and SNR). We reasoned that quality metrics based specifically on the glutamate measurement, such as CRLB and COV for glutamate, might have an advantage over those based on the whole spectrum, such as FWHM and SNR. With regard to the latter two, in our own lab we have consistently found low FWHM to be a better predictor of valid glutamate measurements than high SNR.

In response to a question about this issue during peer review, we searched for and extracted SNR mean and SD values from the 36 mPFC studies included in our meta-analysis. Only 23 studies reported these data, and only 20 used an equivalent method for calculating SNR (the LCModel default method). Applying the same procedure as for the other quality metrics, we identified 14 high quality datasets for SNR (mean minus 2 SD ≥ 13). Six studies were identified as having lower quality SNR values. The 16 studies not reporting SNR were included in the lower-quality subgroup. Moderator analyses showed that effect sizes were not significantly different between lower- and high-quality subgroups for SNR (omnibus model Q = 2.2, df =1, p = .13; heterogeneity: I^2 = 45, p = .002). When studies not reporting SNR were excluded altogether from the moderator analysis, there was trend for mPFC glutamate to be more reduced in the high-quality versus the lower-quality SNR subgroup, but it was not significant with our corrected alpha (omnibus model Q = 3.9, df =1, p = .048; heterogeneity: I^2 = 42, p = .02). Detailed statistics for each SNR subgroup are shown below. In agreement with our expectation, an empirical quality threshold based on SNR was less successful than the other quality metrics at identifying studies sensitive to reduced mPFC glutamate in schizophrenia.

Region	Subgroup	Datasets	Cases	Healthy Controls	Effect Size (95% CI)	P value	Heterogeneity I^2, %	Heterogeneity P value
MPFC	All datasets	36	1022	1064	**-.19 (-.07 to -.32)**	**.003**	48	<.001
	SNR[a] ≥ 13	14	444	521	**-.30 (-.14 to -.47)**	**<.001**	33	.12
	SNR[a] < 13	6	251	231	-.02 (+.32 to -.36)	.91	67	.02
	SNR not stated	16	327	312	-.17 (+.04 to -.38)	.12	42	.04

[a]mean minus 2 standard deviations of SNR values, averaged across patients and controls

Table S1. Excluded studies and reason for exclusion. Abbreviations: glx = glutamate+glutamine, gln = glutamine, HC = healthy control group, MRS = Magnetic Resonance Spectroscopy, NR = Not Reported, SD = Standard Deviation.

Total Count	Count by Reason	Study	Reason for Exclusion
1	1	Atagun et al. (2017) [1]	no glutamate measure (also no gln or glx)
2	2	Bartolomeo et al. (2019) [2]	no glutamate measure (glx)
3	3	Bernier et al. (2016) [3]	no glutamate measure (glx)
4	4	Birur et al. (2020) [4]	no glutamate measure (glx)
5	5	Block et al. (2000) [5]	no glutamate measure (glx)
6	6	Bustillo et al. (2011) [6]	no glutamate measure (glx)
7	7	Bustillo et al. (2017) [7]	no glutamate measure (glx)
8	8	Bustillo et al. (2017) [8]	no glutamate measure (glx)
9	9	Bustillo et al. (2019) [9]	no glutamate measure (glx)
10	10	Cadena et al. (2018) [10]	no glutamate measure (glx)
11	11	Capizzano et al. (2011) [11]	no glutamate measure (glx)
12	12	Cen et al. (2020) [12]	no glutamate measure (glx)
13	13	Chang et al. (2007) [13]	no glutamate measure (glx)
14	14	Chiu et al. (2018) [14]	no glutamate measure (glx)
15	15	Choe et al. (1994) [15]	no glutamate measure (also no gln or glx)
16	16	Choe et al. (1996) [16]	no glutamate measure (also no gln or glx)
17	17	Chouinard et al. (2017) [17]	no glutamate measure (also no gln or glx)
18	18	Conus et al. (2018) [18]	no glutamate measure (also no gln or glx)
19	19	Curcic-Blake et al. (2017) [19]	no glutamate measure (glx)
20	20	Da Silva et al. (2018) [20]	no glutamate measure (also no gln or glx)
21	21	Da Silva et al. (2018) [21]	no glutamate measure (also no gln or glx)
22	22	Da Silva et al. (2019) [22]	no glutamate measure (also no gln or glx)
23	23	Dlabac-de Lange et al. (2017) [23]	no glutamate measure (glx)
24	24	de la Fuente-Sandoval et al. (2015) [24]	no glutamate measure (glx)
25	25	de la Fuente-Sandoval et al. (2018) [25]	no glutamate measure (glx)
26	26	Galinska et al. (2009) [26]	no glutamate measure (glx)
27	27	Galinska-Skok et al. (2018) [27]	no glutamate measure (glx)
28	28	Galinska-Skok et al. (2019) [28]	no glutamate measure (glx)
29	29	Gan et al. (2017) [29]	no glutamate measure (also no gln or glx)
30	30	Goto et al. (2012) [30]	no glutamate measure (glx)
31	31	Grent-'t-Jong et al. (2018) [31]	no glutamate measure (glx)
32	32	Hafizi et al. (2018) [32]	no glutamate measure (also no gln or glx)
33	33	Hasan et al. (2014) [33]	no glutamate measure (glx)
34	34	He et al. (2018) [34]	no glutamate measure (also no gln or glx)
35	35	Huang et al. (2017) [35]	no glutamate measure (glx)
36	36	Huang et al. (2019) [36]	no glutamate measure (glx)
37	37	Hugdahl et al. (2015) [37]	no glutamate measure (glx)

38	38	Hutcheson et al. (2012) [38]	no glutamate measure (glx)
39	39	Jessen et al. (2013) [39]	no glutamate measure (gln and glx)
40	40	Kegeles et al. (2000) [40]	no glutamate measure (glx)
41	41	Kegeles et al. (2012) [41]	no glutamate measure (glx)
42	42	Keshavan et al. (2009) [42]	no glutamate measure (glx)
43	43	Kim et al. (2017) [43]	no glutamate measure (also no gln or glx)
44	44	Kim et al. (2017) [44]	no glutamate measure (also no gln or glx)
45	45	Kirtas et al. (2016) [45]	no glutamate measure (also no gln or glx)
46	46	Klauser et al. (2018) [46]	no glutamate measure (also no gln or glx)
47	47	Kraguljac et al. (2012) [47]	no glutamate measure (glx)
48	48	Kraguljac et al. (2019) [48]	no glutamate measure (glx)
49	49	Larabi et al. (2017) [49]	no glutamate measure (glx)
50	50	Lesh et al. (2019) [50]	no glutamate measure (also no gln or glx)
51	51	Liemburg et al. (2016) [51]	no glutamate measure (glx)
52	52	Liu et al. (2015) [52]	no glutamate measure (also no gln or glx)
53	53	Lotfi et al. (2018) [53]	no glutamate measure (glx)
54	54	Malaspina et al. (2016) [54]	no glutamate measure (also no gln or glx)
55	55	Marenco et al. (2016) [55]	no glutamate measure (also no gln or glx)
56	56	Mazgaj et al. (2016) [56]	no glutamate measure (also no gln or glx)
57	57	McQueen et al. (2020) [57]	no glutamate measure (glx)
58	58	Menschikov et al. (2016) [58]	no glutamate measure (glx)
59	59	Meyer et al. (2016) [59]	no glutamate measure (also no gln or glx)
60	60	Modinos et al. (2018) [60]	no glutamate measure (also no gln or glx)
61	61	Natsubori et al. (2014) [61]	no glutamate measure (glx)
62	62	Ohrmann et al. (2005) [62]	no glutamate measure (glx)
63	63	Ohrmann et al. (2007) [63]	no glutamate measure (glx)
64	64	Ohrmann et al. (2008) [64]	no glutamate measure (glx)
65	65	Ota et al. (2012) [65]	no glutamate measure (glx)
66	66	Ota et al. (2015) [66]	no glutamate measure (glx)
67	67	Piras et al. (2019) [67]	no glutamate measure (also no gln or glx)
68	68	Prasad et al. (2016) [68]	no glutamate measure (also no gln or glx)
69	69	Prasad et al. (2018) [69]	no glutamate measure (also no gln or glx)
70	70	Provenzano et al. (2020) [70]	no glutamate measure (glx)
71	71	Psomiades et al. (2018) [71]	no glutamate measure (also no gln or glx)
72	72	Rauchmann et al. (2020) [72]	no glutamate measure (glx)
73	73	Reid et al. (2016) [73]	no glutamate measure (glx)
74	74	Reyes-Madrigal et al. (2019) [74]	no glutamate measure (also no gln or glx)
75	75	Rogdaki et al. (2019) [75]	no glutamate measure (glx)
76	76	Rowland et al. (2009) [76]	no glutamate measure (glx)
77	77	Rowland et al. (2013) [77]	no glutamate measure (glx)
78	78	Rowland et al. (2016) [78]	no glutamate measure (also no gln or glx)
79	79	Shaw et al. (2020) [79]	no glutamate measure (also no gln or glx)
80	80	Sivaraman et al. (2018) [80]	no glutamate measure (glx)

81	81	Strzelecki et al. (2015) [81]	no glutamate measure (glx)
82	82	Strzelecki et al. (2015) [82]	no glutamate measure (glx)
83	83	Strzelecki et al. (2015) [83]	no glutamate measure (glx)
84	84	Szulc et al. (2004) [84]	no glutamate measure (glx)
85	85	Szulc et al. (2011) [85]	no glutamate measure (glx)
86	86	Tandon et al. (2013) [86]	no glutamate measure (glx)
87	87	Tarumi et al. (2020) [87]	no glutamate measure (glx)
88	88	Tasic et al. (2019) [88]	no glutamate measure (also no gln or glx)
89	89	Thomas et al. (1998) [89]	no glutamate measure (glx)
90	90	Tibbo et al. (2004) [90]	no glutamate measure (glx)
91	91	Ublinskii et al. (2015) [91]	no glutamate measure (also no gln or glx)
92	92	Vingerhoets et al. (2019) [92]	no glutamate measure (also no gln or glx)
93	93	Wang et al. (2016) [93]	no glutamate measure (glx)
94	94	Wijtenburg et al. (2019) [94]	no glutamate measure (also no gln or glx)
95	95	Wood et al. (2007) [95]	no glutamate measure (glx)
96	96	Wood et al. (2008) [96]	no glutamate measure (glx)
97	97	Xia et al. (2018) [97]	no glutamate measure (also no gln or glx)
98	98	Xiang et al. (2019) [98]	no glutamate measure (glx)
99	99	Yamasue et al. (2003) [99]	no glutamate measure (glx)
100	100	Yang et al. (2019) [100]	no glutamate measure (also no gln or glx)
101	101	Yoo et al. (2009) [101]	no glutamate measure (glx)
102	102	Yoon et al. (2020) [102]	no glutamate measure (also no gln or glx)
103	1	Bustillo et al. (2016) [103]	no HC group
104	2	Dempster et al. (2015) [104]	no HC group
105	3	Demro et al. (2017) [105]	no HC group
106	4	Kaur et al. (2019) [106]	no HC group
107	5	Kegeles et al. (2019) [107]	no HC group
108	6	Mouchlianitis et al. (2016) [108]	no HC group
109	7	Nussbaum et al. (2016) [109]	no HC group
110	8	Rowland et al. (2017) [110]	no HC group
111	1	Bustillo et al. (2014) [111]	either means or SDs NR
112	2	Chiappelli et al. (2015) [112]	either means or SDs NR
113	3	Goldstein et al. (2015) [113]	either means or SDs NR
114	4	Limongi et al. (2020) [114]	either means or SDs NR
115	5	Nussbaum et al. (2017) [115]	either means or SDs NR
116	6	Stanley et al. (1996) [116]	either means or SDs NR
117	1	Davies et al. (2019) [117]	intervention study with no baseline
118	2	McQueen et al. (2018) [118]	intervention study with no baseline
119	1	Jelen et al. (2019) [119]	MRS performed during a task
120	2	Taylor et al. (2015) [120]	MRS performed during a task
121	1	da Silva Alves et al. (2011) [121]	22q11.2
122	1	Chiappelli et al. (2016) [122]	participant overlap with other studies
123	2	Chiappelli et al. (2018) [123]	participant overlap with other studies

124	3	Jauhar et al. (2018) [124]	participant overlap with other studies
125	4	Maddock et al. (2018) [125]	participant overlap with other studies
126	5	Merritt et al. (2019) [126]	participant overlap with other studies
127	6	Overbeek et al. (2019) [127]	participant overlap with other studies
128	7	Rowland et al. (2016) [128]	participant overlap with other studies
129	8	Shah et al. (2020) [129]	participant overlap with other studies
130	9	Shukla et al. (2019) [130]	participant overlap with other studies
131	10	Theberge et al. (2002) [131]	participant overlap with other studies
132	1	Ongur et al. (2008) [132]	no internal reference
133	1	Smesny et al. (2015) [133]	multivoxel MRSI acquisition
134	1	Kim et al. (2018) [134]	sex ratio group imbalance
135	1	Girgis et al. (2019) [135]	CT-PRESS sequence
136	1	Bloemen et al. (2011) [136]	high risk participants only
137	2	Bossong et al. (2019) [137]	high risk participants only
138	3	Egerton et al. (2014) [138]	high risk participants only
139	4	Howes et al. (2020) [139]	high risk participants only
140	5	Modinos et al. (2018) [140]	high risk participants only
141	6	Purdon et al. (2008) [141]	high risk participants only
142	7	Stone et al. (2009) [142]	high risk participants only
143	8	Wenneberg et al. (2020) [143]	high risk participants only

Table S2. Included studies of regions for which < 10 datasets are available.

Region	Reference
Thalamus	Aoyama et al. (2011) [165]
Thalamus	Bojesen et al. (2019) [166]
Thalamus	Bustillo et al. (2010) [167]
Thalamus	Egerton et al. (2018) [168]
Thalamus	Legind et al. (2019) [169]
Thalamus	Taylor et al. (2017) [170]
Thalamus	Theberge et al. (2003) [171]
Thalamus	Wang et al. (2019) [151]
Dorsolateral Prefrontal Cortex	Iwata et al. (2019) [145]
Dorsolateral Prefrontal Cortex	Kaminski et al. (2020) [146]
Dorsolateral Prefrontal Cortex	Ragland et al. (2020) [147]
Dorsolateral Prefrontal Cortex	Rusch et al. (2008) [148]
Dorsolateral Prefrontal Cortex	Stanley et al. (2007) [149]
Dorsolateral Prefrontal Cortex	van Elst et al. (2005) [150]
Dorsolateral Prefrontal Cortex	Wang et al. (2019) [151]
Striatum	de la Fuente-Sandoval et al. (2011) [144]
Striatum	Plitman et al. (2016) [161]
Striatum	Plitman et al. (2018) [162]
Striatum	Tayoshi et al. (2009) [163]
Striatum	Thakkar et al. (2017) [158]
Frontal White Matter	Chiappelli et al. (2015) [152]
Frontal White Matter	Lutkenhoff et al. (2010) [153]
Frontal White Matter	Tunc-Skarka et al. (2009) [154]
Frontal White Matter	Wang et al. (2019) [151]
Occipital Cortex	Balz et al. (2018) [156]
Occipital Cortex	Kumar et al. (2020) [155]
Occipital Cortex	Marsman et al. (2014) [157]
Occipital Cortex	Thakkar et al. (2017) [158]
Cerebellum	de la Fuente-Sandoval et al. (2011) [144]
Cerebellum	Piras et al. (2019) [67]
Parietal Cortex	Korenic et al. (2020) [159]
Parietal Cortex	Lee et al. (2018) [160]
Superior Temporal Cortex	Atagun et al. (2015) [164]
Superior Temporal Cortex	Balz et al. (2018) [156]
Inferior Frontal Cortex	Kumar et al. (2020) [155]
Lateral Orbitofrontal Cortex	Wang et al. (2019) [151]
Ventromedial Prefrontal Cortex[a]	Yang et al. (2015) [172]

Table S2. Included studies of regions for which < 10 datasets are available.
The Legind et al. (2019) [149] and Stanley et al. (2007) [157] studies each had 2 datasets. [a]Ventromedial Prefrontal defined as having the midpoint of voxel inferior to the most rostral point of the corpus callosum.

Table S3 Medial Prefrontal Glutamate - Study Characteristics, Glutamate Data, and Quality Metrics.

1st Author/Year/Parameters	HC N	HC Mean	HC STD	PT N	PT Mean	PT STD	Hedges g	95% CRLB	95% FWHM	mean CoV
Bartha 1997 1.5T ST-20	10	11.75	2.75	10	10.3	2	-0.576	NR	NR	0.214
Theberge 2003 4T ST-20	21	15.89	2.81	21	13.31	3.82	-0.755	NR	NR	0.232
Terpstra 2005 4T ST-5	9	10	0.7	13	10.2	0.9	0.233	NR	NR	0.079
Tayoshi 2009 3T ST-18	25	11.5	3.6	30	9.8	2.7	-0.534	NR	NR	0.294
Bustillo 2010 4T ST-20	10	15.82	2.59	14	14.55	1.37	-0.624	0.105	NR	0.129
Lutkenhoff 2010 3T PR-30	21	11.83	3.81	9	8.11	3.34	-0.982	0.229	0.10	0.367
Ongur 2010 3T M-PR-68	17	0.804	0.152	19	0.889	0.21	0.449	0.22	NR	0.213
Shirayama 2010 3T PR-30	18	1.275	0.088	19	1.256	0.15	-0.150	0.082	NR	0.094
Aoyama 2011 4T ST-20	17	15.6	6	15	15.6	5.7	0.000	NR	NR	0.375
Tibbo 2013 3T ST-240	41	7.83	1.8	33	8.05	1.91	0.118	0.149	0.09	0.234
Demjaha 2014 3T PR-30	10	8.62	1.02	14	9.49	2.18	0.467	NR	NR	0.174
Marsman 2014 7T S-LAS-28	18	8.65	1.14	14	8.48	1.34	-0.135	0.042	0.038	0.145
Brandt 2016 7T ST-14	24	9.05	0.72	24	9.16	1.66	0.085	NR	NR	0.130
Gallinat 2016 3T PR-80	27	15.17	1.11	29	14.46	1.56	-0.514	0.158	NR	0.091
Rowland 2016 7T ST-14	29	8.1	0.67	27	7.9	0.85	-0.259	NR	0.0405	0.095
Xin 2016 3T SPEC-6	33	14.18	0.98	25	13.47	1.59	-0.548	0.03	0.043	0.094
Chen 2017 3T M-PR-68	24	6.54	1.99	24	6.07	2.48	-0.206	NR	NR	0.356
Taylor 2017 7T ST-10	18	10	1.3	15	10.7	1.2	0.543	NR	NR	0.121
Wijtenburg-1 2017 3T ST-6.5	54	9.54	0.7	48	9.25	0.8	-0.384	0.065	0.066	0.080
Wijtenburg-2 2017 3T ST-6.5	39	8.71	0.8	47	8.16	1	-0.596	0.077	0.062	0.107
Chiappelli 2018 3T ST-6.5	21	13.62	0.96	20	12.9	1.25	-0.635	0.063	0.0585	0.084
Egerton 2018 3T PR-30	60	1.339	0.137	70	1.335	0.164	-0.028	0.088	0.06	0.113
Kumar 2018 7T ST-17	45	6.21	0.81	27	6.01	0.66	-0.261	NR	0.085	0.120
Posperilis 2018 7T ST-15	20	1.33	0.14	20	1.29	0.1	-0.322	0.025	0.058	0.091
Rigucci 2018 3T SPEC-6	33	14.2	0.9	35	13.28	1.52	-0.719	0.03	NR	0.089
Bojesen 2019 3T PR-30	36	1.55	0.1	37	1.51	0.14	-0.325	0.064	0.098	0.079
Borgan 2019 3T PR-30	65	13	2.2	46	13.2	2	0.094	NR	0.0586	0.160
Hjelmervik 2019 3T PR-35	33	16.55	1.69	33	17.09	1.8	0.306	NR	0.09	0.104
Iwata 2019 3T PR-35	26	15.98	1.74	74	16.94	1.66	0.568	NR	0.0678	0.103
Legind-1 2019 3T PR-30	49	10.36	1.03	28	10.44	1.1	0.075	0.072	0.075	0.102
Legind-2 2019 3T PR-30	36	10.26	0.9	22	10.43	1.65	0.136	0.074	0.07	0.123
Pillinger 2019 3T PR-30	18	1.33	0.21	19	1.25	0.18	-0.401	0.099	NR	0.151
Reid 2019 7T ST-5	21	6.93	0.5	21	6.57	0.5	-0.706	0.029	0.038	0.074

1st Author/Year/Parameters	HC N	HC Mean	HC STD	PT N	PT Mean	PT STD	Hedges g	95% CRLB	95% FWHM	mean CoV
Wang 2019 7T ST-14	87	8.16	0.57	75	7.83	0.65	-0.540	0.03	0.042	0.076
Dempster 2020 7T LAS-100	27	8.35	2.3	26	8.51	2.05	0.072	0.066	NR	0.258
Korenic 2020 3T PR-30	22	13.3	1.3	19	13.2	1.2	-0.078	NR	NR	0.094

Table S3: Studies ordered by year of publication. First author, year, field strength, sequence and TE are shown in first column. ST = STEAM; PR = PRESS; LAS = LASER; M-PR = MEGA-PRESS; SPEC = SPECIAL; HC = healthy controls; PT = patients; N = sample size; Mean = mean glutamate value; STD = standard deviation of glutamate values; 95% CRLB = mean + 2SD CRLB values averaged across patient and control groups; 95% FWHM = mean + 2SD FWHM values in PPM averaged across patient and control groups; mean COV = coefficient of variation of glutamate values (STD/mean) averaged across patient and control groups.

Table S4 Hippocampal Glutamate - Study Characteristics, Glutamate Data, and Quality Metrics

1st Author/Year/Parameters	HC N	HC Mean	HC STD	PT N	PT Mean	PT STD	Hedges g	95% CRLB	95% FWHM	mean CoV
Bartha 1999 1.5T ST-20	11	7.58	1.67	11	6.83	2.32	-0.356	NR	NR	0.280
van Elst 2005 2T PR-30	16	2.37	0.8	8	4.34	2.83	1.100	NR	NR	0.495
Olbrich 2008 2T PR-30	32	2.37	1.13	9	3.91	0.87	1.395	NR	NR	0.350
Rusch 2008 2T PR-30	12	2.67	0.7	14	3.56	1.44	0.742	NR	NR	0.333
Lutkenhoff 2010 3T PR-30	21	9.26	5.67	9	7.54	1.9	-0.341	0.264	0.10	0.432
Nenadic 2015 3T PR-30	42	9.878	2.628	18	10.095	2.53	0.082	NR	NR	0.258
Stan 2015 3T M-PR-70	16	0.88	0.08	18	0.82	0.09	-0.685	0.063	NR	0.100
Gallinat 2016 3T PR-80	29	10.42	1.53	29	12.1	1.47	1.105	NR	NR	0.134
Singh 2018 3T PR-33	28	1.24	0.21	28	1.15	0.16	-0.475	NR	NR	0.154
Korenic 2020 3T PR-30	21	8.5	1.3	19	8.2	1.3	-0.226	NR	NR	0.156
Shakory 2020 3T PR-35	31	10.51	1.4	10	10.12	1.15	-0.284	0.09	.092	0.123

Table S4: Studies ordered by year of publication. First author, year, field strength, sequence and TE are shown in first column. ST = STEAM; PR = PRESS; M-PR = MEGA-PRESS; HC = healthy controls; PT = patients; N = sample size; Mean = mean glutamate value; STD = standard deviation of glutamate values; 95% CRLB = mean + 2SD CRLB values averaged across patient and control groups; 95% FWHM = mean + 2SD FWHM values in PPM averaged across patient and control groups; mean COV = coefficient of variation of glutamate values (STD/mean) averaged across patient and control groups.

Moderator effects

Meta-regression analysis showed no effect of either field strength or log TE. The distributions of these regressors, however, were very limited. There were no studies above 3T, and 8 of the 11 studies used TE between 30 and 35 ms. Three datasets were categorized as ≥ 80% unmedicated and 6 datasets as all medicated (2 datasets excluded). Medication status did not significantly moderate effect size (omnibus model Q = 2.2, df =1, p = .14; heterogeneity: I^2 = 75, p < .001). Similarly, 4 datasets were categorized as recent onset and 7 as chronic. Neither phase of illness nor mean patient age significantly moderated effect size (omnibus model Q = 0.0, df =1, NS; heterogeneity: I^2 = 80, p = .001; and omnibus model Q = 2.5, df =1, p = .114; heterogeneity: I^2 = 74, p = .001, respectively).

Table S5 Thalamic Glutamate - Study Characteristics, Glutamate Data, and Quality Metrics

1st Author/Year/Parameters	HC N	HC Mean	HC STD	PT N	PT Mean	PT STD	Hedges g	95% CRLB	95% FWHM	mean CoV
Theberge 2003 4T ST-20	19	13.11	1.74	19	12.88	1.37	-0.144	NR	NR	0.120
Bustillo 2010 4T ST-20	10	11.5	4.26	12	12.29	3.38	0.200	0.275	NR	0.323
Aoyama 2011 4T ST-20	17	13.89	2.69	16	13.71	1.79	-0.076	NR	NR	0.348
Taylor 2017 7T ST-10	18	7.4	0.60	15	7.4	1.00	0.0	NR	NR	0.108
Egerton 2018 3T PR-30	60	1.09	.15	70	1.09	.20	0.0	0.165	0.075	0.1623
Bojesen 2019 3T PR-30	32	1.22	.15	38	1.29	.15	0.461	0.13	0.057	0.120
Legind-1 2019 3T PR-30	52	7.07	.84	23	7.55	.84	0.565	0.185	0.075	0.115
Legind-2 2019 3T PR-30	36	7.34	.92	22	7.45	1.13	0.108	0.155	0.075	0.139
Wang 2019 7T ST-14	74	6.36	0.54	66	6.24	0.63	-0.204	0.10	0.0705	0.093

Table S5: Studies ordered by year of publication. First author, year, field strength, sequence and TE are shown in first column. ST = STEAM; PR = PRESS; HC = healthy controls; PT = patients; N = sample size; Mean = mean glutamate value; STD = standard deviation of glutamate values; 95% CRLB = mean + 2SD CRLB values averaged across patient and control groups; 95% FWHM = mean + 2SD FWHM values in PPM averaged across patient and control groups; mean COV = coefficient of variation of glutamate values (STD/mean) averaged across patient and control groups.

Table S6 Dorsolateral Prefrontal Glutamate - Study Characteristics, Glutamate Data, and Quality Metrics

1st Author/Year/Parameters	HC N	HC Mean	HC STD	PT N	PT Mean	PT STD	Hedges g	95% CRLB	95% FWHM	mean CoV
van Elst 2005 2T PR-30	33	2.9	0.7	21	7.49	8.4	0.876	NR	NR	0.682
Stanley-1 2007 1.5T ST-20	27	6.91	1.31	8	5.82	.92	-0.858	NR	0.080	0.174
Stanley-2 2007 1.5T ST-20	34	6.45	1.48	10	6.42	1.11	-0.021	NR	0.080	0.202
Rusch 2008 2T PR-30	22	3.04	0.64	20	3.82	1.43	0.702	NR	NR	0.292
Iwata 2019 3T PR-35	26	13.94	1.32	21	13.55	1.6	-0.264	0.058	0.077	0.106
Wang 2019 7T ST-14	84	6.65	0.52	72	6.41	0.75	-0.375	0.044	0.049	0.162
Kaminski 2020 3T PR-80	35	8.22	0.9	55	7.92	1.1	-0.289	NR	NR	0.124
Ragland 2020 3T PR-80	49	0.906	0.091	38	0.894	0.104	-0.123	0.072	0.056	0.108

Table S6: Studies ordered by year of publication. First author, year, field strength, sequence and TE are shown in first column. ST = STEAM; PR = PRESS; HC = healthy controls; PT = patients; N = sample size; Mean = mean glutamate value; STD = standard deviation of glutamate values; 95% CRLB = mean + 2SD CRLB values averaged across patient and control groups; 95% FWHM = mean + 2SD FWHM values in PPM averaged across patient and control groups; mean COV = coefficient of variation of glutamate values (STD/mean) averaged across patient and control group

Figure S1: PRISMA Flow Diagram[173]

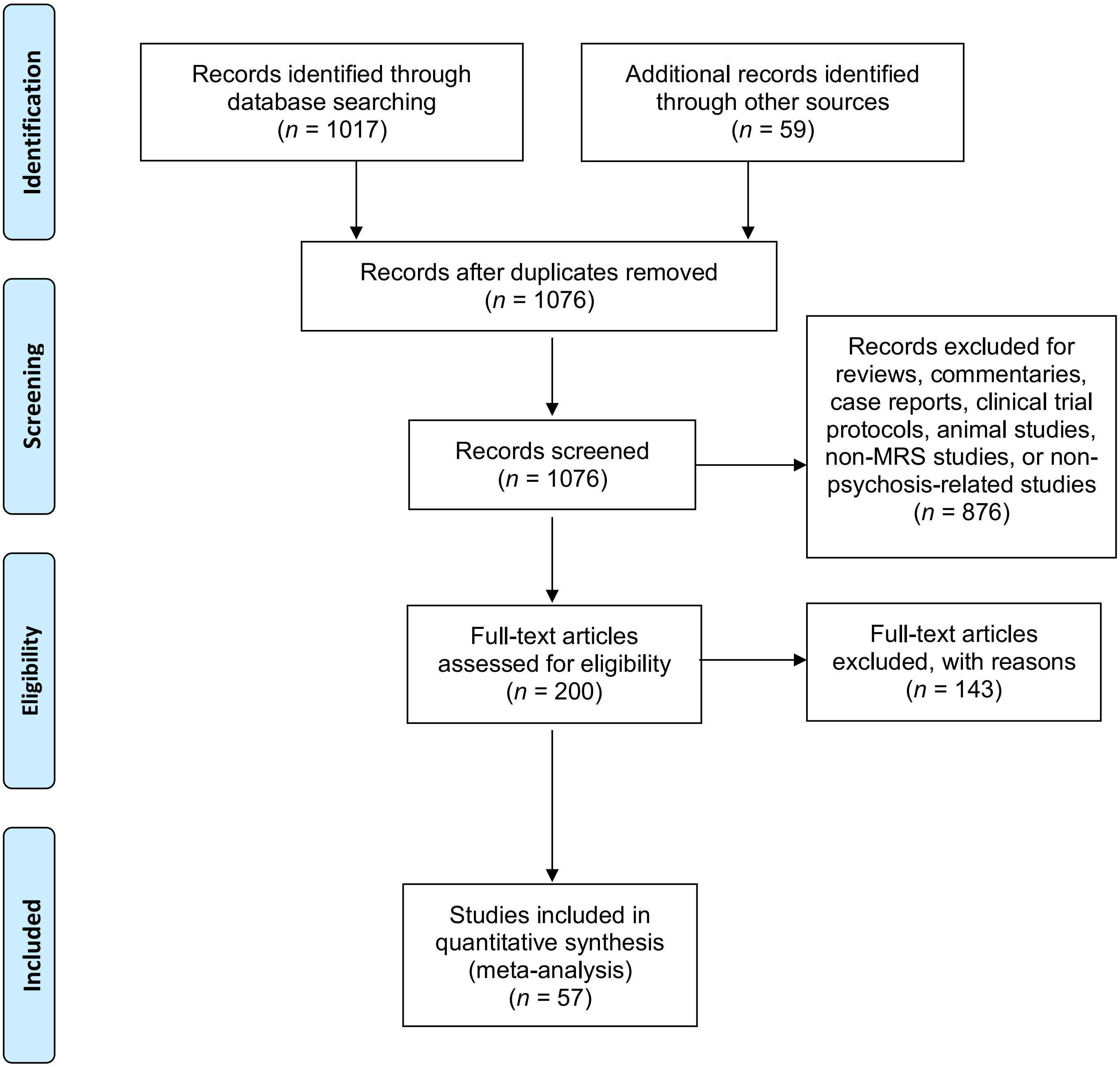

Figure S2
Hippocampal Glutamate

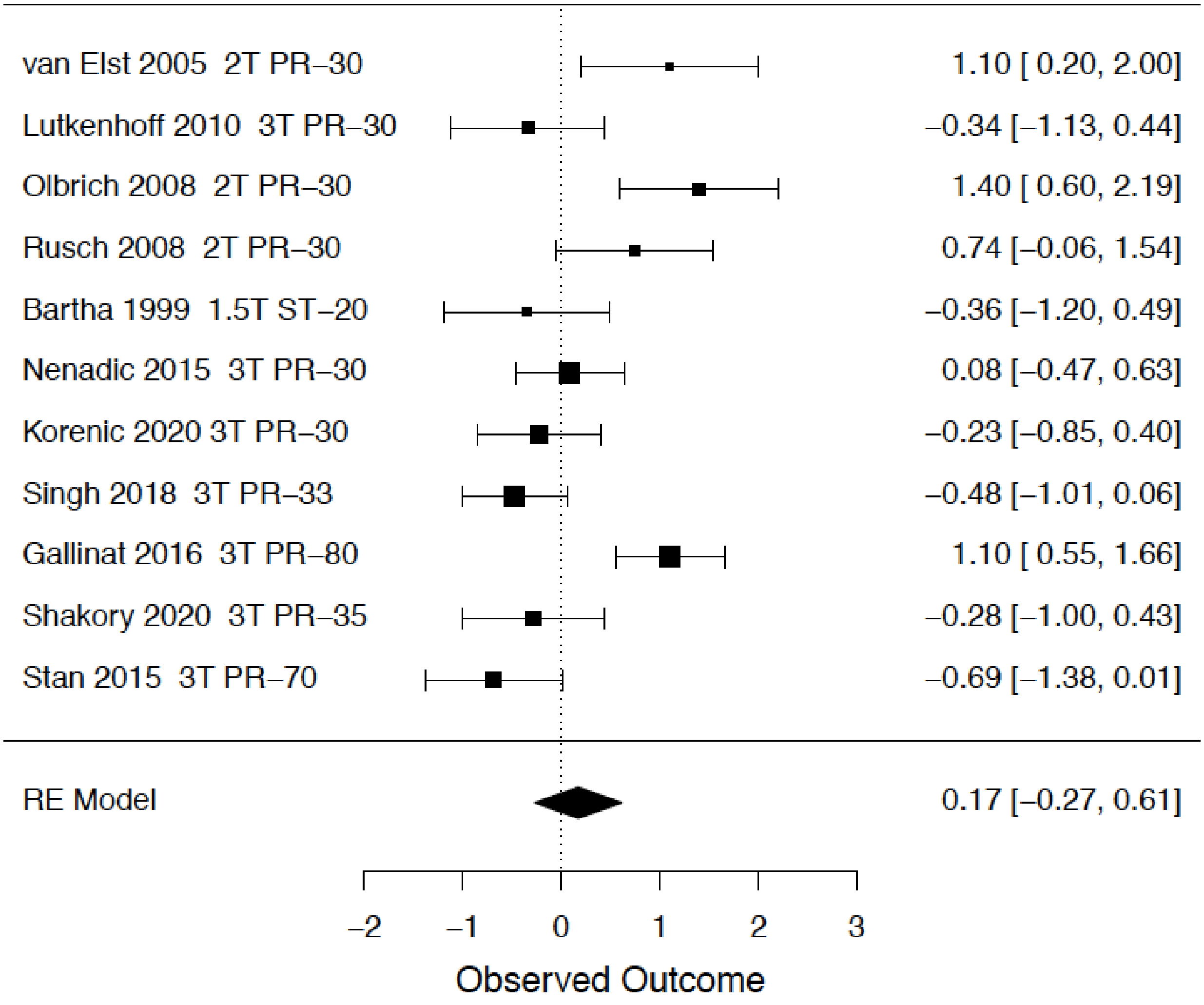

Figure S2: Meta-analysis forest plot for 11 datasets reporting hippocampal glutamate in schizophrenia patients and healthy control subjects. Datasets are listed in order of coefficient of variation (COV) of glutamate averaged across patient and control groups, with lowest COV at bottom. First author, year, field strength, sequence and TE are listed at left. Hedge's *g* and 95% CI are at center and right. ST = STEAM; PR = PRESS; RE = random effects.

Figure S3
Thalamic Glutamate

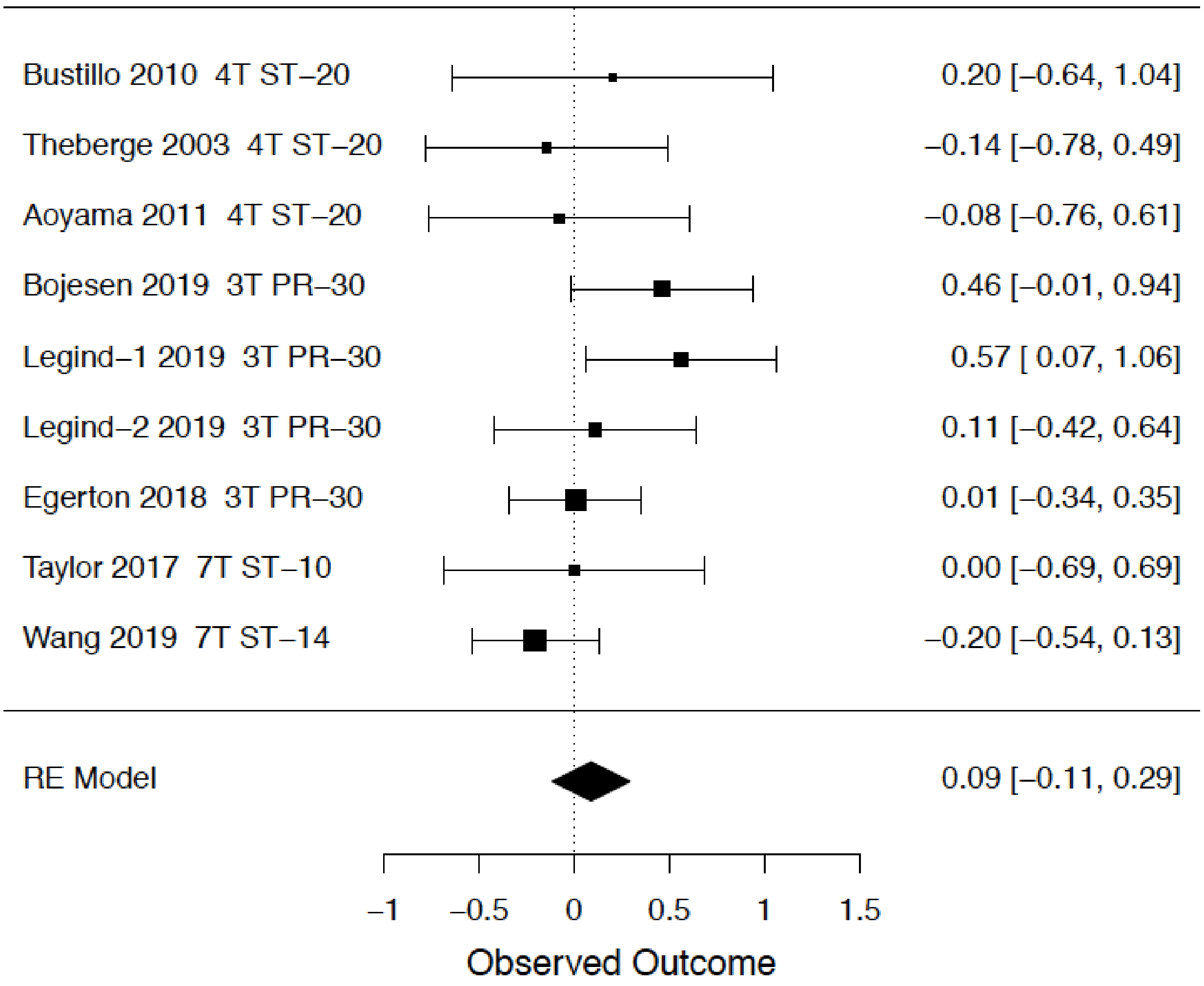

Figure S3. Meta-analysis forest plot for 9 datasets reporting thalamic glutamate in schizophrenia patients and healthy control subjects. Datasets are listed in order of coefficient of variation (COV) of glutamate averaged across patient and control groups, with lowest COV at bottom. First author, year, field strength, sequence and TE are listed at left. Hedge's *g* and 95% CI are at center and right. ST = STEAM; PR = PRESS; RE = random effects.

Figure S4
Dorsolateral PFC Glutamate

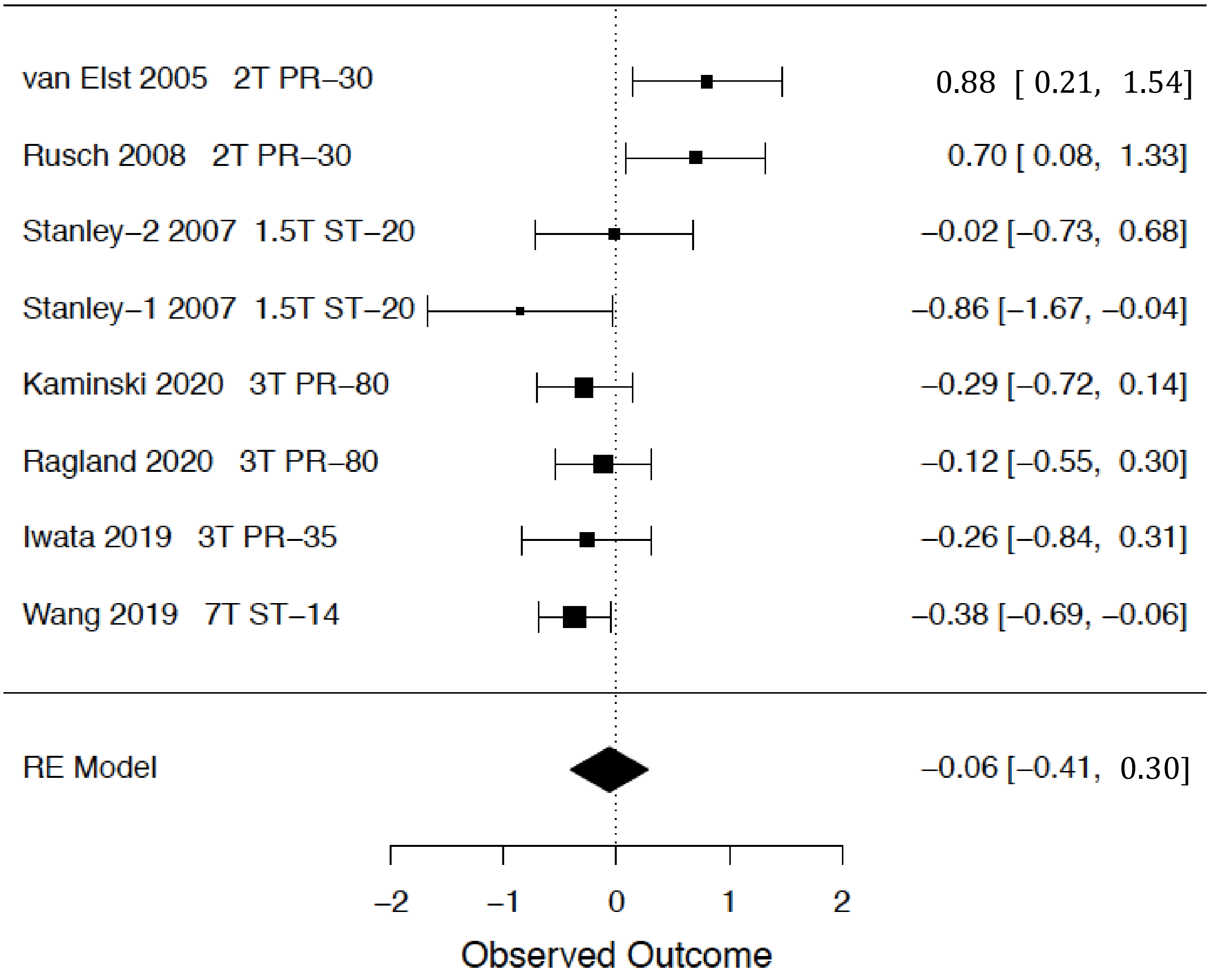

Figure S4. Meta-analysis forest plot for 8 datasets reporting dorsolateral PFC glutamate in schizophrenia patients and healthy control subjects. Datasets are listed in order of coefficient of variation (COV) of glutamate averaged across patient and control groups, with lowest COV at bottom. First author, year, field strength, sequence and TE are listed at left. Hedge's *g* and 95% CI are at center and right. ST = STEAM; PR = PRESS; RE = random effects.

Figure S5
Striatal Glutamate

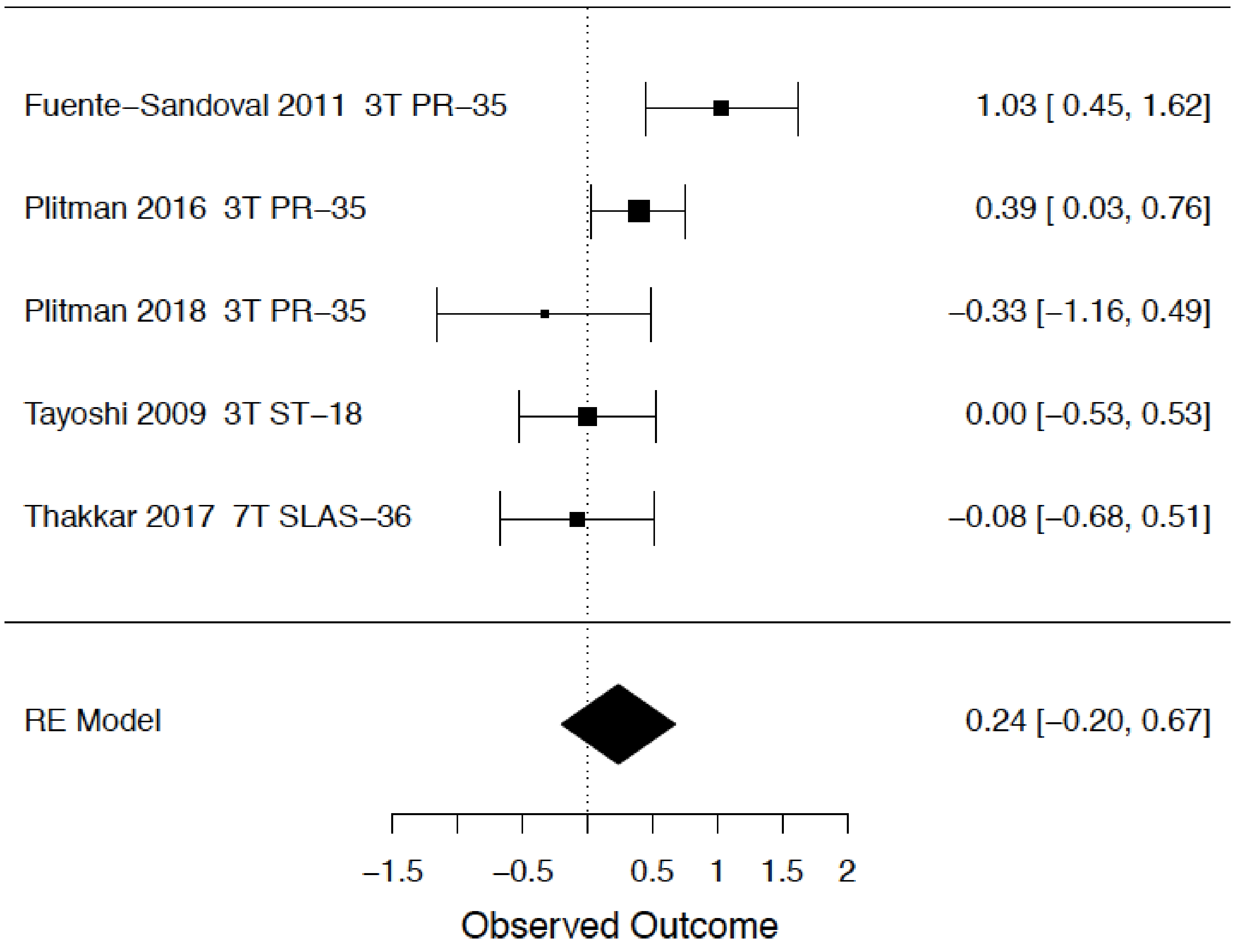

Figure S5. Exploratory meta-analysis forest plot for 5 datasets reporting striatal glutamate in schizophrenia patients and healthy control subjects. Datasets are listed in order of coefficient of variation (COV) of glutamate averaged across patient and control groups, with lowest COV at bottom. First author, year, field strength, sequence and TE are listed at left. Hedge's *g* and 95% CI are at center and right. ST = STEAM; PR = PRESS; SLAS = Semi-LASER; RE = random effects.

Figure S6
Frontal White Matter Glutamate

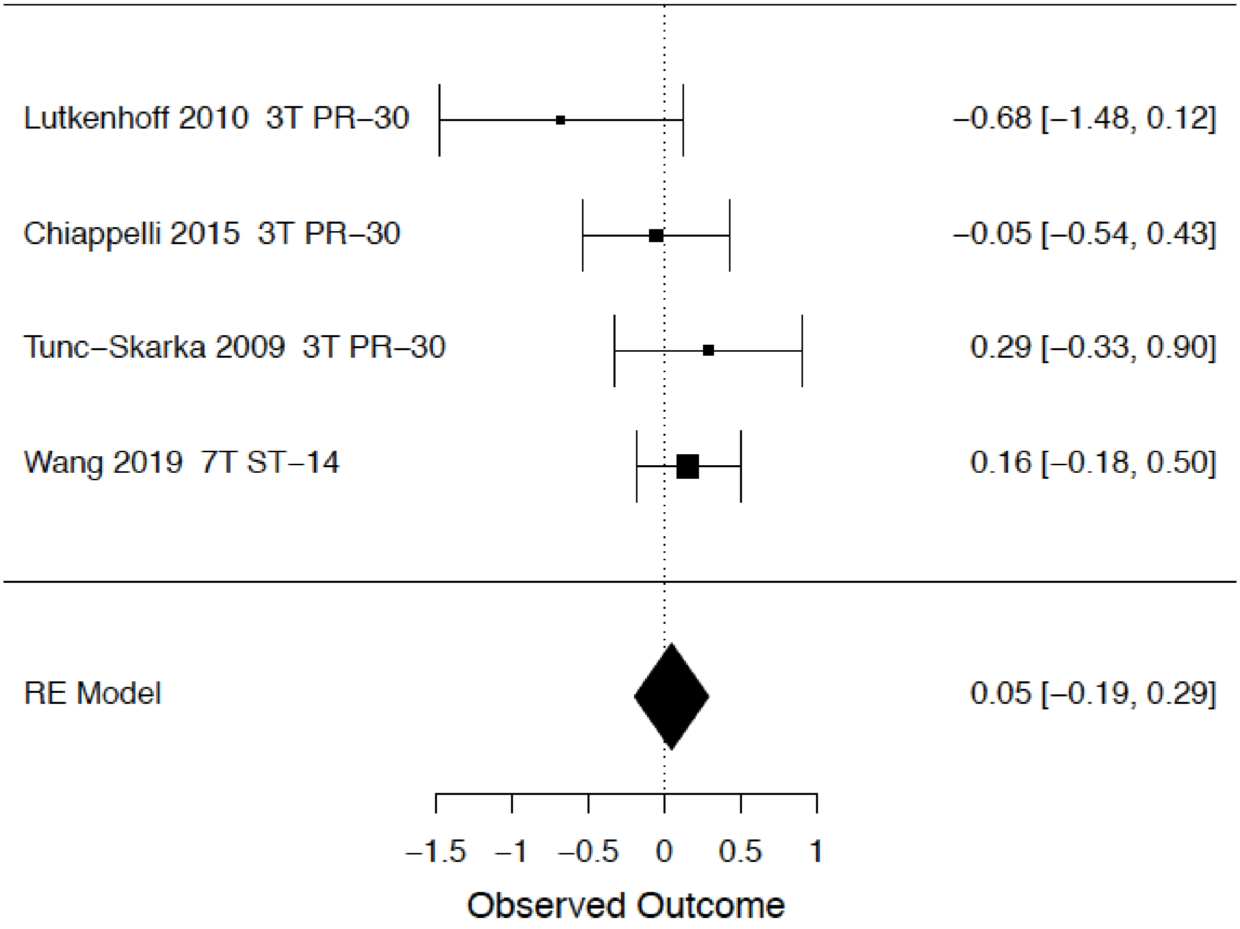

Figure S6. Exploratory meta-analysis forest plot for 4 datasets reporting frontal white matter glutamate in schizophrenia patients and healthy control subjects. Datasets are listed in order of coefficient of variation (COV) of glutamate averaged across patient and control groups, with lowest COV at bottom. First author, year, field strength, sequence and TE are listed at left. Hedge's *g* and 95% CI are at center and right. ST = STEAM; PR = PRESS; RE = random effects.

Figure S7
Occipital Cortex Glutamate

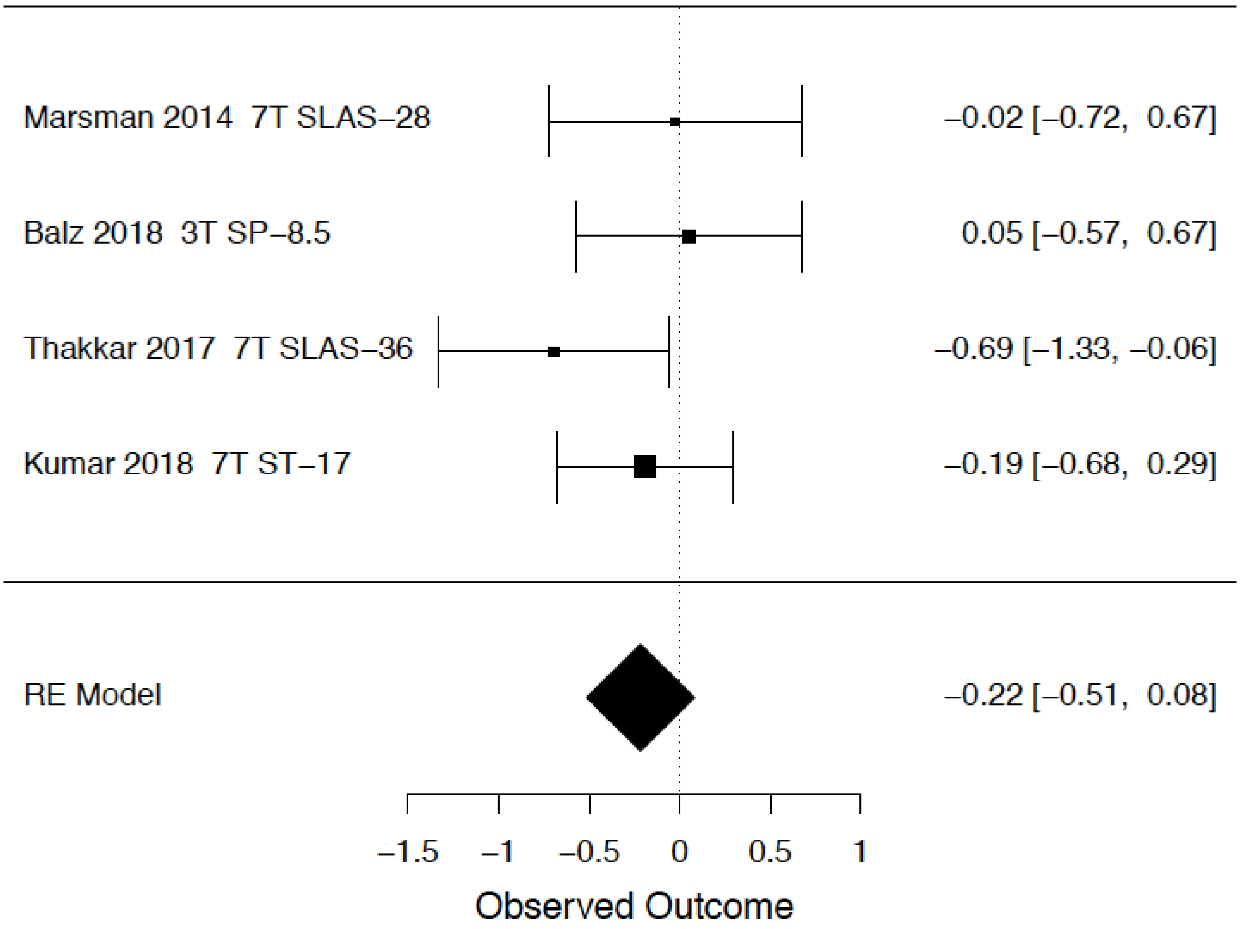

Figure S7. Exploratory meta-analysis forest plot for 4 datasets reporting occipital cortex glutamate in schizophrenia patients and healthy control subjects. Datasets are listed in order of coefficient of variation (COV) of glutamate averaged across patient and control groups, with lowest COV at bottom. First author, year, field strength, sequence and TE are listed at left. Hedge's *g* and 95% CI are at center and right. ST = STEAM; PR = PRESS; SLAS = Semi-LASER; RE = random effects.

Supplemental References

1. Atagun MI, Sikoglu EM, Soykan C, et al. Perisylvian GABA levels in schizophrenia and bipolar disorder. *Neurosci Lett.* 2017;637:70-74.

2. Bartolomeo LA, Wright AM, Ma RE, et al. Relationship of auditory electrophysiological responses to magnetic resonance spectroscopy metabolites in Early Phase Psychosis. *Int J Psychophysiol.* 2019;145:15-22.

3. Bernier D, Bartha R, McAllindon D, et al. Illness versus substance use effects on the frontal white matter in early phase schizophrenia: A 4Tesla (1)H-MRS study. *Schizophr Res.* 2016;175(1-3):4-11.

4. Birur B, Kraguljac NV, VerHoef L, et al. Neurometabolic correlates of 6 and 16 weeks of treatment with risperidone in medication-naive first-episode psychosis patients. *Transl Psychiatry.* 2020;10(1):15.

5. Block W, Bayer TA, Tepest R, et al. Decreased frontal lobe ratio of N-acetyl aspartate to choline in familial schizophrenia: a proton magnetic resonance spectroscopy study. *Neurosci Lett.* 2000;289(2):147-151.

6. Bustillo JR, Chen H, Gasparovic C, et al. Glutamate as a marker of cognitive function in schizophrenia: a proton spectroscopic imaging study at 4 Tesla. *Biol Psychiatry.* 2011;69(1):19-27.

7. Bustillo JR, Jones T, Chen H, et al. Glutamatergic and Neuronal Dysfunction in Gray and White Matter: A Spectroscopic Imaging Study in a Large Schizophrenia Sample. *Schizophr Bull.* 2017;43(3):611-619.

8. Bustillo JR, Patel V, Jones T, et al. Risk-Conferring Glutamatergic Genes and Brain Glutamate Plus Glutamine in Schizophrenia. *Front Psychiatry.* 2017;8:79.

9. Bustillo JR, Jones T, Qualls C, et al. Proton magnetic resonance spectroscopic imaging of gray and white matter in bipolar-I and schizophrenia. *J Affect Disord.* 2019;246:745-753.

10. Cadena EJ, White DM, Kraguljac NV, et al. A Longitudinal Multimodal Neuroimaging Study to Examine Relationships Between Resting State Glutamate and Task Related BOLD Response in Schizophrenia. *Front Psychiatry.* 2018;9:632.

11. Capizzano AA, Toscano JL, Ho BC. Magnetic resonance spectroscopy of limbic structures displays metabolite differences in young unaffected relatives of schizophrenia probands. *Schizophr Res.* 2011;131(1-3):4-10.

12. Cen H, Xu J, Yang Z, et al. Neurochemical and brain functional changes in the ventromedial prefrontal cortex of first-episode psychosis patients: A combined

functional magnetic resonance imaging-proton magnetic resonance spectroscopy study. *Aust N Z J Psychiatry.* 2020;54(5):519-527.

13. Chang L, Friedman J, Ernst T, Zhong K, Tsopelas ND, Davis K. Brain metabolite abnormalities in the white matter of elderly schizophrenic subjects: implication for glial dysfunction. *Biol Psychiatry.* 2007;62(12):1396-1404.

14. Chiu PW, Lui SSY, Hung KSY, et al. In vivo gamma-aminobutyric acid and glutamate levels in people with first-episode schizophrenia: A proton magnetic resonance spectroscopy study. *Schizophr Res.* 2018;193:295-303.

15. Choe BY, Kim KT, Suh TS, et al. 1H magnetic resonance spectroscopy characterization of neuronal dysfunction in drug-naive, chronic schizophrenia. *Acad Radiol.* 1994;1(3):211-216.

16. Choe BY, Suh TS, Shinn KS, Lee CW, Lee C, Paik IH. Observation of metabolic changes in chronic schizophrenia after neuroleptic treatment by in vivo hydrogen magnetic resonance spectroscopy. *Invest Radiol.* 1996;31(6):345-352.

17. Chouinard VA, Kim SY, Valeri L, et al. Brain bioenergetics and redox state measured by (31)P magnetic resonance spectroscopy in unaffected siblings of patients with psychotic disorders. *Schizophr Res.* 2017;187:11-16.

18. Conus P, Seidman LJ, Fournier M, et al. N-acetylcysteine in a Double-Blind Randomized Placebo-Controlled Trial: Toward Biomarker-Guided Treatment in Early Psychosis. *Schizophr Bull.* 2018;44(2):317-327.

19. Curcic-Blake B, Bais L, Sibeijn-Kuiper A, et al. Glutamate in dorsolateral prefrontal cortex and auditory verbal hallucinations in patients with schizophrenia: A (1)H MRS study. *Prog Neuropsychopharmacol Biol Psychiatry.* 2017;78:132-139.

20. Da Silva T, Hafizi S, Andreazza AC, et al. Glutathione, the Major Redox Regulator, in the Prefrontal Cortex of Individuals at Clinical High Risk for Psychosis. *Int J Neuropsychopharmacol.* 2018;21(4):311-318.

21. Da Silva T, Wu A, Laksono I, et al. Mitochondrial function in individuals at clinical high risk for psychosis. *Sci Rep.* 2018;8(1):6216.

22. Da Silva T, Hafizi S, Rusjan PM, et al. GABA levels and TSPO expression in people at clinical high risk for psychosis and healthy volunteers: a PET-MRS study. *J Psychiatry Neurosci.* 2019;44(2):111-119.

23. Dlabac-de Lange JJ, Liemburg EJ, Bais L, et al. Effect of Bilateral Prefrontal rTMS on Left Prefrontal NAA and Glx Levels in Schizophrenia Patients with Predominant Negative Symptoms: An Exploratory Study. *Brain Stimul.* 2017;10(1):59-64.

24. de la Fuente-Sandoval C, Reyes-Madrigal F, Mao X, et al. Cortico-Striatal GABAergic and Glutamatergic Dysregulations in Subjects at Ultra-High Risk for Psychosis Investigated with Proton Magnetic Resonance Spectroscopy. *Int J Neuropsychopharmacol.* 2015;19(3):pyv105.

25. de la Fuente-Sandoval C, Reyes-Madrigal F, Mao X, et al. Prefrontal and Striatal Gamma-Aminobutyric Acid Levels and the Effect of Antipsychotic Treatment in First-Episode Psychosis Patients. *Biol Psychiatry.* 2018;83(6):475-483.

26. Galinska B, Szulc A, Tarasow E, et al. Duration of untreated psychosis and proton magnetic resonance spectroscopy (1H-MRS) findings in first-episode schizophrenia. *Med Sci Monit.* 2009;15(2):CR82-88.

27. Galinska-Skok B, Malus A, Konarzewska B, et al. Choline Compounds of the Frontal Lobe and Temporal Glutamatergic System in Bipolar and Schizophrenia Proton Magnetic Resonance Spectroscopy Study. *Dis Markers.* 2018;2018:3654894.

28. Galinska-Skok B, Szulc A, Malus A, et al. Proton magnetic resonance spectroscopy changes in a longitudinal schizophrenia study: a pilot study in eleven patients. *Neuropsychiatr Dis Treat.* 2019;15:839-847.

29. Gan JL, Duan HF, Cheng ZX, et al. Neuroprotective Effect of Modified Electroconvulsive Therapy for Schizophrenia: A Proton Magnetic Resonance Spectroscopy Study. *J Nerv Ment Dis.* 2017;205(6):480-486.

30. Goto N, Yoshimura R, Kakeda S, et al. Six-month treatment with atypical antipsychotic drugs decreased frontal-lobe levels of glutamate plus glutamine in early-stage first-episode schizophrenia. *Neuropsychiatr Dis Treat.* 2012;8:119-122.

31. Grent-'t-Jong T, Gross J, Goense J, et al. Resting-state gamma-band power alterations in schizophrenia reveal E/I-balance abnormalities across illness-stages. *Elife.* 2018;7.

32. Hafizi S, Da Silva T, Meyer JH, et al. Interaction between TSPO-a neuroimmune marker-and redox status in clinical high risk for psychosis: a PET-MRS study. *Neuropsychopharmacology.* 2018;43(8):1700-1705.

33. Hasan A, Wobrock T, Falkai P, et al. Hippocampal integrity and neurocognition in first-episode schizophrenia: a multidimensional study. *World J Biol Psychiatry.* 2014;15(3):188-199.

34. He Y, Kosciolek T, Tang J, et al. Gut microbiome and magnetic resonance spectroscopy study of subjects at ultra-high risk for psychosis may support the membrane hypothesis. *Eur Psychiatry.* 2018;53:37-45.

35. Huang ML, Khoh TT, Lu SJ, et al. Relationships between dorsolateral prefrontal cortex metabolic change and cognitive impairment in first-episode neuroleptic-naive schizophrenia patients. *Medicine (Baltimore).* 2017;96(25):e7228.

36. Huang M, Guo W, Lu S, et al. The relationship between the alterations in metabolite levels in the dorsolateral prefrontal cortex and clinical symptoms of patients with first-episode schizophrenia: a one year follow-up study. *Oncotarget.* 2019;10(5):606-615.

37. Hugdahl K, Craven AR, Nygard M, et al. Glutamate as a mediating transmitter for auditory hallucinations in schizophrenia: a (1)H MRS study. *Schizophr Res.* 2015;161(2-3):252-260.

38. Hutcheson NL, Reid MA, White DM, et al. Multimodal analysis of the hippocampus in schizophrenia using proton magnetic resonance spectroscopy and functional magnetic resonance imaging. *Schizophr Res.* 2012;140(1-3):136-142.

39. Jessen F, Fingerhut N, Sprinkart AM, et al. N-acetylaspartylglutamate (NAAG) and N-acetylaspartate (NAA) in patients with schizophrenia. *Schizophr Bull.* 2013;39(1):197-205.

40. Kegeles LS, Shungu DC, Anjilvel S, et al. Hippocampal pathology in schizophrenia: magnetic resonance imaging and spectroscopy studies. *Psychiatry Res.* 2000;98(3):163-175.

41. Kegeles LS, Mao X, Stanford AD, et al. Elevated prefrontal cortex gamma-aminobutyric acid and glutamate-glutamine levels in schizophrenia measured in vivo with proton magnetic resonance spectroscopy. *Arch Gen Psychiatry.* 2012;69(5):449-459.

42. Keshavan MS, Dick RM, Diwadkar VA, Montrose DM, Prasad KM, Stanley JA. Striatal metabolic alterations in non-psychotic adolescent offspring at risk for schizophrenia: a (1)H spectroscopy study. *Schizophr Res.* 2009;115(1):88-93.

43. Kim SY, Cohen BM, Chen X, et al. Redox Dysregulation in Schizophrenia Revealed by in vivo NAD+/NADH Measurement. *Schizophr Bull.* 2017;43(1):197-204.

44. Kim JH, Kim JH, Park PW, et al. Body and liver fat content and adipokines in schizophrenia: a magnetic resonance imaging and spectroscopy study. *Psychopharmacology (Berl).* 2017;234(12):1923-1932.

45. Kirtas D, Karadag RF, Balci Sengul MC, Kiroglu Y. 1H-magnetic resonance spectroscopy in first episode and chronic schizophrenia patients. *Turk J Med Sci.* 2016;46(3):862-871.

46. Klauser P, Xin L, Fournier M, et al. N-acetylcysteine add-on treatment leads to an improvement of fornix white matter integrity in early psychosis: a double-blind randomized placebo-controlled trial. *Transl Psychiatry.* 2018;8(1):220.

47. Kraguljac NV, Reid MA, White DM, den Hollander J, Lahti AC. Regional decoupling of N-acetyl-aspartate and glutamate in schizophrenia. *Neuropsychopharmacology.* 2012;37(12):2635-2642.

48. Kraguljac NV, Morgan CJ, Reid MA, et al. A longitudinal magnetic resonance spectroscopy study investigating effects of risperidone in the anterior cingulate cortex and hippocampus in schizophrenia. *Schizophr Res.* 2019;210:239-244.

49. Larabi DI, Liemburg EJ, Pijnenborg GH, et al. Association between prefrontal N-acetylaspartate and insight in psychotic disorders. *Schizophr Res.* 2017;179:112-118.

50. Lesh TA, Maddock RJ, Howell A, et al. Extracellular free water and glutathione in first-episode psychosis-a multimodal investigation of an inflammatory model for psychosis. *Mol Psychiatry.* 2019.

51. Liemburg E, Sibeijn-Kuiper A, Bais L, et al. Prefrontal NAA and Glx Levels in Different Stages of Psychotic Disorders: a 3T 1H-MRS Study. *Sci Rep.* 2016;6:21873.

52. Liu W, Yu H, Jiang B, et al. The predictive value of baseline NAA/Cr for treatment response of first-episode schizophrenia: A (1)H MRS study. *Neurosci Lett.* 2015;600:199-205.

53. Lotfi M, Ghaderian Jahromi M, Firoozabadi A, Razeghian Jahromi L. Effect of Adjuvant Electroconvulsive Therapy Compared to Antipsychotic Medication Alone on the Brain Metabolites of Patients with Chronic Schizophrenia: A Proton Magnetic Resonance Spectroscopy Study. *Iran J Psychiatry.* 2018;13(3):215-221.

54. Malaspina D, Kranz TM, Heguy A, et al. Prefrontal neuronal integrity predicts symptoms and cognition in schizophrenia and is sensitive to genetic heterogeneity. *Schizophr Res.* 2016;172(1-3):94-100.

55. Marenco S, Meyer C, Kuo S, et al. Prefrontal GABA Levels Measured With Magnetic Resonance Spectroscopy in Patients With Psychosis and Unaffected Siblings. *Am J Psychiatry.* 2016;173(5):527-534.

56. Mazgaj R, Tal A, Goetz R, et al. Hypo-metabolism of the rostral anterior cingulate cortex associated with working memory impairment in 18 cases of schizophrenia. *Brain Imaging Behav.* 2016;10(1):115-123.

57. McQueen G, Lay A, Lally J, et al. Effect of single dose N-acetylcysteine administration on resting state functional connectivity in schizophrenia. *Psychopharmacology (Berl).* 2020;237(2):443-451.

58. Menschikov PE, Semenova NA, Ublinskiy MV, et al. (1)H-MRS and MEGA-PRESS pulse sequence in the study of balance of inhibitory and excitatory neurotransmitters in the human brain of ultra-high risk of schizophrenia patients. *Dokl Biochem Biophys.* 2016;468(1):168-172.

59. Meyer EJ, Kirov, II, Tal A, et al. Metabolic Abnormalities in the Hippocampus of Patients with Schizophrenia: A 3D Multivoxel MR Spectroscopic Imaging Study at 3T. *AJNR Am J Neuroradiol.* 2016;37(12):2273-2279.

60. Modinos G, Simsek F, Azis M, et al. Prefrontal GABA levels, hippocampal resting perfusion and the risk of psychosis. *Neuropsychopharmacology.* 2018;43(13):2652-2659.

61. Natsubori T, Inoue H, Abe O, et al. Reduced frontal glutamate + glutamine and N-acetylaspartate levels in patients with chronic schizophrenia but not in those at clinical high risk for psychosis or with first-episode schizophrenia. *Schizophr Bull.* 2014;40(5):1128-1139.

62. Ohrmann P, Siegmund A, Suslow T, et al. Evidence for glutamatergic neuronal dysfunction in the prefrontal cortex in chronic but not in first-episode patients with schizophrenia: a proton magnetic resonance spectroscopy study. *Schizophr Res.* 2005;73(2-3):153-157.

63. Ohrmann P, Siegmund A, Suslow T, et al. Cognitive impairment and in vivo metabolites in first-episode neuroleptic-naive and chronic medicated schizophrenic patients: a proton magnetic resonance spectroscopy study. *J Psychiatr Res.* 2007;41(8):625-634.

64. Ohrmann P, Kugel H, Bauer J, et al. Learning potential on the WCST in schizophrenia is related to the neuronal integrity of the anterior cingulate cortex as measured by proton magnetic resonance spectroscopy. *Schizophr Res.* 2008;106(2-3):156-163.

65. Ota M, Ishikawa M, Sato N, et al. Glutamatergic changes in the cerebral white matter associated with schizophrenic exacerbation. *Acta Psychiatr Scand.* 2012;126(1):72-78.

66. Ota M, Wakabayashi C, Sato N, et al. Effect of L-theanine on glutamatergic function in patients with schizophrenia. *Acta Neuropsychiatr.* 2015;27(5):291-296.

67. Piras F, Piras F, Banaj N, et al. Cerebellar GABAergic correlates of cognition-mediated verbal fluency in physiology and schizophrenia. *Acta Psychiatr Scand.* 2019;139(6):582-594.

68. Prasad KM, Burgess AM, Keshavan MS, Nimgaonkar VL, Stanley JA. Neuropil pruning in Early-Course Schizophrenia: Immunological, Clinical, and Neurocognitive Correlates. *Biol Psychiatry Cogn Neurosci Neuroimaging.* 2016;1(6):528-538.

69. Prasad KM, Chowdari KV, D'Aiuto LA, Iyengar S, Stanley JA, Nimgaonkar VL. Neuropil contraction in relation to Complement C4 gene copy numbers in independent cohorts of adolescent-onset and young adult-onset schizophrenia patients-a pilot study. *Transl Psychiatry.* 2018;8(1):134.

70. Provenzano FA, Guo J, Wall MM, et al. Hippocampal Pathology in Clinical High-Risk Patients and the Onset of Schizophrenia. *Biol Psychiatry.* 2020;87(3):234-242.

71. Psomiades M, Mondino M, Fonteneau C, et al. N-Acetyl-Aspartate in the dorsolateral prefrontal cortex in men with schizophrenia and auditory verbal hallucinations: A 1.5 T Magnetic Resonance Spectroscopy Study. *Sci Rep.* 2018;8(1):4133.

72. Rauchmann BS, Ghaseminejad F, Keeser D, et al. The impact of endurance training and table soccer on brain metabolites in schizophrenia. *Brain Imaging Behav.* 2020;14(2):515-526.

73. Reid MA, White DM, Kraguljac NV, Lahti AC. A combined diffusion tensor imaging and magnetic resonance spectroscopy study of patients with schizophrenia. *Schizophr Res.* 2016;170(2-3):341-350.

74. Reyes-Madrigal F, Leon-Ortiz P, Mao X, Mora-Duran R, Shungu DC, de la Fuente-Sandoval C. Striatal Glutathione in First-episode Psychosis Patients Measured In Vivo with Proton Magnetic Resonance Spectroscopy. *Arch Med Res.* 2019;50(4):207-213.

75. Rogdaki M, Hathway P, Gudbrandsen M, et al. Glutamatergic function in a genetic high-risk group for psychosis: A proton magnetic resonance spectroscopy study in individuals with 22q11.2 deletion. *Eur Neuropsychopharmacol.* 2019;29(12):1333-1342.

76. Rowland LM, Spieker EA, Francis A, Barker PB, Carpenter WT, Buchanan RW. White matter alterations in deficit schizophrenia. *Neuropsychopharmacology.* 2009;34(6):1514-1522.

77. Rowland LM, Kontson K, West J, et al. In vivo measurements of glutamate, GABA, and NAAG in schizophrenia. *Schizophr Bull.* 2013;39(5):1096-1104.

78. Rowland LM, Krause BW, Wijtenburg SA, et al. Medial frontal GABA is lower in older schizophrenia: a MEGA-PRESS with macromolecule suppression study. *Mol Psychiatry.* 2016;21(2):198-204.

79. Shaw AD, Knight L, Freeman TCA, et al. Oscillatory, Computational, and Behavioral Evidence for Impaired GABAergic Inhibition in Schizophrenia. *Schizophr Bull.* 2020;46(2):345-353.

80. Sivaraman S, Kraguljac NV, White DM, Morgan CJ, Gonzales SS, Lahti AC. Neurometabolic abnormalities in the associative striatum in antipsychotic-naive first episode psychosis patients. *Psychiatry Res Neuroimaging.* 2018;281:101-106.

81. Strzelecki D, Podgorski M, Kaluzynska O, et al. Supplementation of Antipsychotic Treatment with the Amino Acid Sarcosine Influences Proton Magnetic Resonance Spectroscopy Parameters in Left Frontal White Matter in Patients with Schizophrenia. *Nutrients.* 2015;7(10):8767-8782.

82. Strzelecki D, Podgorski M, Kaluzynska O, et al. Adding Sarcosine to Antipsychotic Treatment in Patients with Stable Schizophrenia Changes the Concentrations of Neuronal and Glial Metabolites in the Left Dorsolateral Prefrontal Cortex. *Int J Mol Sci.* 2015;16(10):24475-24489.

83. Strzelecki D, Grzelak P, Podgorski M, et al. Comparison of Metabolite Concentrations in the Left Dorsolateral Prefrontal Cortex, the Left Frontal White Matter, and the Left Hippocampus in Patients in Stable Schizophrenia Treated with Antipsychotics with or without Antidepressants. (1)H-NMR Spectroscopy Study. *Int J Mol Sci.* 2015;16(10):24387-24402.

84. Szulc A, Galinska B, Tarasow E, et al. Glutamatergic system dysfunction in schizophrenia. A proton magnetic resonance spectroscopy (1H MRS) study. *Pol J Radiol.* 2004;69(1):33-36.

85. Szulc A, Galinska B, Tarasow E, et al. Proton magnetic resonance spectroscopy study of brain metabolite changes after antipsychotic treatment. *Pharmacopsychiatry.* 2011;44(4):148-157.

86. Tandon N, Bolo NR, Sanghavi K, et al. Brain metabolite alterations in young adults at familial high risk for schizophrenia using proton magnetic resonance spectroscopy. *Schizophr Res.* 2013;148(1-3):59-66.

87. Tarumi R, Tsugawa S, Noda Y, et al. Levels of glutamatergic neurometabolites in patients with severe treatment-resistant schizophrenia: a proton magnetic resonance spectroscopy study. *Neuropsychopharmacology.* 2020;45(4):632-640.

88. Tasic L, Larcerda ALT, Pontes JGM, et al. Peripheral biomarkers allow differential diagnosis between schizophrenia and bipolar disorder. *J Psychiatr Res.* 2019;119:67-75.

89. Thomas MA, Ke Y, Levitt J, et al. Preliminary study of frontal lobe 1H MR spectroscopy in childhood-onset schizophrenia. *J Magn Reson Imaging.* 1998;8(4):841-846.

90. Tibbo P, Hanstock C, Valiakalayil A, Allen P. 3-T proton MRS investigation of glutamate and glutamine in adolescents at high genetic risk for schizophrenia. *Am J Psychiatry.* 2004;161(6):1116-1118.

91. Ublinskii MV, Semenova NA, Lukovkina OV, et al. Characteristics of Diffusion in the Corticospinal Tract of Patients with Early Stage of Schizophrenia: Diffusion Tensor Magnetic Resonance Imaging. *Bull Exp Biol Med.* 2015;159(1):29-31.

92. Vingerhoets C, Bakker G, Schrantee A, et al. Influence of muscarinic M1 receptor antagonism on brain choline levels and functional connectivity in medication-free subjects with psychosis: A placebo controlled, cross-over study. *Psychiatry Res Neuroimaging.* 2019;290:5-13.

93. Wang J, Tang Y, Zhang T, et al. Reduced gamma-Aminobutyric Acid and Glutamate+Glutamine Levels in Drug-Naive Patients with First-Episode Schizophrenia but Not in Those at Ultrahigh Risk. *Neural Plast.* 2016;2016:3915703.

94. Wijtenburg SA, Kapogiannis D, Korenic SA, et al. Brain insulin resistance and altered brain glucose are related to memory impairments in schizophrenia. *Schizophr Res.* 2019;208:324-330.

95. Wood SJ, Yucel M, Wellard RM, et al. Evidence for neuronal dysfunction in the anterior cingulate of patients with schizophrenia: a proton magnetic resonance spectroscopy study at 3 T. *Schizophr Res.* 2007;94(1-3):328-331.

96. Wood SJ, Berger GE, Wellard RM, et al. A 1H-MRS investigation of the medial temporal lobe in antipsychotic-naive and early-treated first episode psychosis. *Schizophr Res.* 2008;102(1-3):163-170.

97. Xia M, Wang J, Sheng J, et al. Effect of Electroconvulsive Therapy on Medial Prefrontal gamma-Aminobutyric Acid Among Schizophrenia Patients: A Proton Magnetic Resonance Spectroscopy Study. *J ECT.* 2018;34(4):227-232.

98. Xiang Q, Xu J, Wang Y, et al. Modular Functional-Metabolic Coupling Alterations of Frontoparietal Network in Schizophrenia Patients. *Front Neurosci.* 2019;13:40.

99. Yamasue H, Fukui T, Fukuda R, et al. Drug-induced parkinsonism in relation to choline-containing compounds measured by 1H-MR spectroscopy in putamen of chronically medicated patients with schizophrenia. *Int J Neuropsychopharmacol.* 2003;6(4):353-360.

100. Yang YS, Maddock RJ, Lee J, et al. Brain glutathione levels and age at onset of illness in chronic schizophrenia. *Acta Neuropsychiatr.* 2019;31(6):343-347.

101. Yoo SY, Yeon S, Choi CH, et al. Proton magnetic resonance spectroscopy in subjects with high genetic risk of schizophrenia: investigation of anterior

cingulate, dorsolateral prefrontal cortex and thalamus. *Schizophr Res.* 2009;111(1-3):86-93.

102. Yoon JH, Maddock RJ, DongBo Cui E, et al. Reduced in vivo visual cortex GABA in schizophrenia, a replication in a recent onset sample. *Schizophr Res.* 2020;215:217-222.

103. Bustillo JR, Rediske N, Jones T, Rowland LM, Abbott C, Wijtenburg SA. Reproducibility of phase rotation stimulated echo acquisition mode at 3T in schizophrenia: Emphasis on glutamine. *Magn Reson Med.* 2016;75(2):498-502.

104. Dempster K, Norman R, Theberge J, Densmore M, Schaefer B, Williamson P. Glutamatergic metabolite correlations with neuropsychological tests in first episode schizophrenia. *Psychiatry Res.* 2015;233(2):180-185.

105. Demro C, Rowland L, Wijtenburg SA, et al. Glutamatergic metabolites among adolescents at risk for psychosis. *Psychiatry Res.* 2017;257:179-185.

106. Kaur M, Chitty KM, Lagopoulos J, Hickie IB, Duffy SL, Hermens DF. Elucidating the glutamatergic processes underlying mismatch negativity deficits in early stage bipolar disorder and schizophrenia: A combined (1)H-MRS and EEG study. *J Psychiatr Res.* 2019;113:83-89.

107. Kegeles LS, Ciarleglio A, Leon-Ortiz P, et al. An imaging-based risk calculator for prediction of conversion to psychosis in clinical high-risk individuals using glutamate (1)H MRS. *Schizophr Res.* 2019.

108. Mouchlianitis E, Bloomfield MA, Law V, et al. Treatment-Resistant Schizophrenia Patients Show Elevated Anterior Cingulate Cortex Glutamate Compared to Treatment-Responsive. *Schizophr Bull.* 2016;42(3):744-752.

109. Nussbaum LA, Hogea LM, Andreescu NI, Gradinaru RC, Puiu M, Todica A. The prognostic and clinical significance of neuroimagistic and neurobiological vulnerability markers in correlation with the molecular pharmacogenetic testing in psychoses and ultra high-risk categories. *Rom J Morphol Embryol.* 2016;57(3):959-967.

110. Rowland LM, Demyanovich HK, Wijtenburg SA, et al. Antigliadin Antibodies (AGA IgG) Are Related to Neurochemistry in Schizophrenia. *Front Psychiatry.* 2017;8:104.

111. Bustillo JR, Chen H, Jones T, et al. Increased glutamine in patients undergoing long-term treatment for schizophrenia: a proton magnetic resonance spectroscopy study at 3 T. *JAMA Psychiatry.* 2014;71(3):265-272.

112. Chiappelli J, Rowland LM, Wijtenburg SA, et al. Evaluation of Myo-Inositol as a Potential Biomarker for Depression in Schizophrenia. *Neuropsychopharmacology.* 2015;40(9):2157-2164.

113. Goldstein ME, Anderson VM, Pillai A, Kydd RR, Russell BR. Glutamatergic neurometabolites in clozapine-responsive and -resistant schizophrenia. *Int J Neuropsychopharmacol.* 2015;18(6).

114. Limongi R, Jeon P, Mackinley M, et al. Glutamate and Dysconnection in the Salience Network: Neurochemical, Effective Connectivity, and Computational Evidence in Schizophrenia. *Biol Psychiatry.* 2020.

115. Nussbaum LA, Hogea LM, Chiriac DV, et al. The effect of neurobiological changes in the brain of children with schizophrenia, ultra high-risk for psychosis and epilepsy: clinical correlations with EEG and neuroimagistic abnormalities. *Rom J Morphol Embryol.* 2017;58(4):1435-1446.

116. Stanley JA, Williamson PC, Drost DJ, et al. An in vivo proton magnetic resonance spectroscopy study of schizophrenia patients. *Schizophr Bull.* 1996;22(4):597-609.

117. Davies C, Rutigliano G, De Micheli A, et al. Neurochemical effects of oxytocin in people at clinical high risk for psychosis. *Eur Neuropsychopharmacol.* 2019;29(5):601-615.

118. McQueen G, Lally J, Collier T, et al. Effects of N-acetylcysteine on brain glutamate levels and resting perfusion in schizophrenia. *Psychopharmacology (Berl).* 2018;235(10):3045-3054.

119. Jelen LA, King S, Horne CM, Lythgoe DJ, Young AH, Stone JM. Functional magnetic resonance spectroscopy in patients with schizophrenia and bipolar affective disorder: Glutamate dynamics in the anterior cingulate cortex during a working memory task. *Eur Neuropsychopharmacol.* 2019;29(2):222-234.

120. Taylor R, Neufeld RW, Schaefer B, et al. Functional magnetic resonance spectroscopy of glutamate in schizophrenia and major depressive disorder: anterior cingulate activity during a color-word Stroop task. *NPJ Schizophr.* 2015;1:15028.

121. da Silva Alves F, Boot E, Schmitz N, et al. Proton magnetic resonance spectroscopy in 22q11 deletion syndrome. *PLoS One.* 2011;6(6):e21685.

122. Chiappelli J, Postolache TT, Kochunov P, et al. Tryptophan Metabolism and White Matter Integrity in Schizophrenia. *Neuropsychopharmacology.* 2016;41(10):2587-2595.

123. Chiappelli J, Rowland LM, Notarangelo FM, et al. Salivary kynurenic acid response to psychological stress: inverse relationship to cortical glutamate in schizophrenia. *Neuropsychopharmacology.* 2018;43(8):1706-1711.

124. Jauhar S, McCutcheon R, Borgan F, et al. The relationship between cortical glutamate and striatal dopamine in first-episode psychosis: a cross-sectional

multimodal PET and magnetic resonance spectroscopy imaging study. *Lancet Psychiatry.* 2018;5(10):816-823.

125. Maddock RJ, Caton MD, Ragland JD. Estimating glutamate and Glx from GABA-optimized MEGA-PRESS: Off-resonance but not difference spectra values correspond to PRESS values. *Psychiatry Res Neuroimaging.* 2018;279:22-30.

126. Merritt K, Perez-Iglesias R, Sendt KV, et al. Remission from antipsychotic treatment in first episode psychosis related to longitudinal changes in brain glutamate. *NPJ Schizophr.* 2019;5(1):12.

127. Overbeek G, Gawne TJ, Reid MA, et al. Relationship Between Cortical Excitation and Inhibition and Task-Induced Activation and Deactivation: A Combined Magnetic Resonance Spectroscopy and Functional Magnetic Resonance Imaging Study at 7T in First-Episode Psychosis. *Biol Psychiatry Cogn Neurosci Neuroimaging.* 2019;4(2):121-130.

128. Rowland LM, Summerfelt A, Wijtenburg SA, et al. Frontal Glutamate and gamma-Aminobutyric Acid Levels and Their Associations With Mismatch Negativity and Digit Sequencing Task Performance in Schizophrenia. *JAMA Psychiatry.* 2016;73(2):166-174.

129. Shah P, Plitman E, Iwata Y, et al. Glutamatergic neurometabolites and cortical thickness in treatment-resistant schizophrenia: Implications for glutamate-mediated excitotoxicity. *J Psychiatr Res.* 2020;124:151-158.

130. Shukla DK, Wijtenburg SA, Chen H, et al. Anterior Cingulate Glutamate and GABA Associations on Functional Connectivity in Schizophrenia. *Schizophr Bull.* 2019;45(3):647-658.

131. Theberge J, Bartha R, Drost DJ, et al. Glutamate and glutamine measured with 4.0 T proton MRS in never-treated patients with schizophrenia and healthy volunteers. *Am J Psychiatry.* 2002;159(11):1944-1946.

132. Ongur D, Jensen JE, Prescot AP, et al. Abnormal glutamatergic neurotransmission and neuronal-glial interactions in acute mania. *Biol Psychiatry.* 2008;64(8):718-726.

133. Smesny S, Gussew A, Biesel NJ, et al. Glutamatergic dysfunction linked to energy and membrane lipid metabolism in frontal and anterior cingulate cortices of never treated first-episode schizophrenia patients. *Schizophr Res.* 2015;168(1-2):322-329.

134. Kim SY, Kaufman MJ, Cohen BM, et al. In Vivo Brain Glycine and Glutamate Concentrations in Patients With First-Episode Psychosis Measured by Echo Time-Averaged Proton Magnetic Resonance Spectroscopy at 4T. *Biol Psychiatry.* 2018;83(6):484-491.

135. Girgis RR, Baker S, Mao X, et al. Effects of acute N-acetylcysteine challenge on cortical glutathione and glutamate in schizophrenia: A pilot in vivo proton magnetic resonance spectroscopy study. *Psychiatry Res.* 2019;275:78-85.

136. Bloemen OJ, Gleich T, de Koning MB, et al. Hippocampal glutamate levels and striatal dopamine D(2/3) receptor occupancy in subjects at ultra high risk of psychosis. *Biol Psychiatry.* 2011;70(1):e1-2; author reply e3.

137. Bossong MG, Antoniades M, Azis M, et al. Association of Hippocampal Glutamate Levels With Adverse Outcomes in Individuals at Clinical High Risk for Psychosis. *JAMA Psychiatry.* 2019;76(2):199-207.

138. Egerton A, Stone JM, Chaddock CA, et al. Relationship between brain glutamate levels and clinical outcome in individuals at ultra high risk of psychosis. *Neuropsychopharmacology.* 2014;39(12):2891-2899.

139. Howes OD, Bonoldi I, McCutcheon RA, et al. Glutamatergic and dopaminergic function and the relationship to outcome in people at clinical high risk of psychosis: a multi-modal PET-magnetic resonance brain imaging study. *Neuropsychopharmacology.* 2020;45(4):641-648.

140. Modinos G, Simsek F, Horder J, et al. Cortical GABA in Subjects at Ultra-High Risk of Psychosis: Relationship to Negative Prodromal Symptoms. *Int J Neuropsychopharmacol.* 2018;21(2):114-119.

141. Purdon SE, Valiakalayil A, Hanstock CC, Seres P, Tibbo P. Elevated 3T proton MRS glutamate levels associated with poor Continuous Performance Test (CPT-0X) scores and genetic risk for schizophrenia. *Schizophr Res.* 2008;99(1-3):218-224.

142. Stone JM, Day F, Tsagaraki H, et al. Glutamate dysfunction in people with prodromal symptoms of psychosis: relationship to gray matter volume. *Biol Psychiatry.* 2009;66(6):533-539.

143. Wenneberg C, Nordentoft M, Rostrup E, et al. Cerebral Glutamate and Gamma-Aminobutyric Acid Levels in Individuals at Ultra-high Risk for Psychosis and the Association With Clinical Symptoms and Cognition. *Biol Psychiatry Cogn Neurosci Neuroimaging.* 2020;5(6):569-579.

144. de la Fuente-Sandoval C, Leon-Ortiz P, Favila R, et al. Higher levels of glutamate in the associative-striatum of subjects with prodromal symptoms of schizophrenia and patients with first-episode psychosis. *Neuropsychopharmacology.* 2011;36(9):1781-1791.

145. Iwata Y, Nakajima S, Plitman E, et al. Glutamatergic Neurometabolite Levels in Patients With Ultra-Treatment-Resistant Schizophrenia: A Cross-Sectional 3T Proton Magnetic Resonance Spectroscopy Study. *Biol Psychiatry.* 2019;85(7):596-605.

146. Kaminski J, Gleich T, Fukuda Y, et al. Association of Cortical Glutamate and Working Memory Activation in Patients With Schizophrenia: A Multimodal Proton Magnetic Resonance Spectroscopy and Functional Magnetic Resonance Imaging Study. *Biol Psychiatry.* 2020;87(3):225-233.

147. Ragland JD, Maddock RJ, Hurtado MY, et al. Disrupted GABAergic facilitation of working memory performance in people with schizophrenia. *Neuroimage Clin.* 2020;25:102127.

148. Rusch N, Tebartz van Elst L, Valerius G, et al. Neurochemical and structural correlates of executive dysfunction in schizophrenia. *Schizophr Res.* 2008;99(1-3):155-163.

149. Stanley JA, Vemulapalli M, Nutche J, et al. Reduced N-acetyl-aspartate levels in schizophrenia patients with a younger onset age: a single-voxel 1H spectroscopy study. *Schizophr Res.* 2007;93(1-3):23-32.

150. van Elst LT, Valerius G, Buchert M, et al. Increased prefrontal and hippocampal glutamate concentration in schizophrenia: evidence from a magnetic resonance spectroscopy study. *Biol Psychiatry.* 2005;58(9):724-730.

151. Wang AM, Pradhan S, Coughlin JM, et al. Assessing Brain Metabolism With 7-T Proton Magnetic Resonance Spectroscopy in Patients With First-Episode Psychosis. *JAMA Psychiatry.* 2019;76(3):314-323.

152. Chiappelli J, Hong LE, Wijtenburg SA, et al. Alterations in frontal white matter neurochemistry and microstructure in schizophrenia: implications for neuroinflammation. *Transl Psychiatry.* 2015;5:e548.

153. Lutkenhoff ES, van Erp TG, Thomas MA, et al. Proton MRS in twin pairs discordant for schizophrenia. *Mol Psychiatry.* 2010;15(3):308-318.

154. Tunc-Skarka N, Weber-Fahr W, Hoerst M, Meyer-Lindenberg A, Zink M, Ende G. MR spectroscopic evaluation of N-acetylaspartate's T2 relaxation time and concentration corroborates white matter abnormalities in schizophrenia. *Neuroimage.* 2009;48(3):525-531.

155. Kumar J, Liddle EB, Fernandes CC, et al. Glutathione and glutamate in schizophrenia: a 7T MRS study. *Mol Psychiatry.* 2020;25(4):873-882.

156. Balz J, Roa Romero Y, Keil J, et al. Glutamate Concentration in the Superior Temporal Sulcus Relates to Neuroticism in Schizophrenia. *Front Psychol.* 2018;9:578.

157. Marsman A, Mandl RC, Klomp DW, et al. GABA and glutamate in schizophrenia: a 7 T (1)H-MRS study. *Neuroimage Clin.* 2014;6:398-407.

158. Thakkar KN, Rosler L, Wijnen JP, et al. 7T Proton Magnetic Resonance Spectroscopy of Gamma-Aminobutyric Acid, Glutamate, and Glutamine Reveals Altered Concentrations in Patients With Schizophrenia and Healthy Siblings. *Biol Psychiatry.* 2017;81(6):525-535.

159. Korenic SA, Klingaman EA, Wickwire EM, et al. Sleep quality is related to brain glutamate and symptom severity in schizophrenia. *J Psychiatr Res.* 2020;120:14-20.

160. Lee J, Yoon YB, Wijtenburg SA, et al. Lower glutamate level in temporo-parietal junction may predict a better response to tDCS in schizophrenia. *Schizophr Res.* 2018;201:422-423.

161. Plitman E, de la Fuente-Sandoval C, Reyes-Madrigal F, et al. Elevated Myo-Inositol, Choline, and Glutamate Levels in the Associative Striatum of Antipsychotic-Naive Patients With First-Episode Psychosis: A Proton Magnetic Resonance Spectroscopy Study With Implications for Glial Dysfunction. *Schizophr Bull.* 2016;42(2):415-424.

162. Plitman E, Chavez S, Nakajima S, et al. Striatal neurometabolite levels in patients with schizophrenia undergoing long-term antipsychotic treatment: A proton magnetic resonance spectroscopy and reliability study. *Psychiatry Res Neuroimaging.* 2018;273:16-24.

163. Tayoshi S, Sumitani S, Taniguchi K, et al. Metabolite changes and gender differences in schizophrenia using 3-Tesla proton magnetic resonance spectroscopy (1H-MRS). *Schizophr Res.* 2009;108(1-3):69-77.

164. Atagun MI, Sikoglu EM, Can SS, et al. Investigation of Heschl's gyrus and planum temporale in patients with schizophrenia and bipolar disorder: a proton magnetic resonance spectroscopy study. *Schizophr Res.* 2015;161(2-3):202-209.

165. Aoyama N, Theberge J, Drost DJ, et al. Grey matter and social functioning correlates of glutamatergic metabolite loss in schizophrenia. *Br J Psychiatry.* 2011;198(6):448-456.

166. Bojesen KB, Ebdrup BH, Jessen K, et al. Treatment response after 6 and 26 weeks is related to baseline glutamate and GABA levels in antipsychotic-naive patients with psychosis. *Psychol Med.* 2019:1-12.

167. Bustillo JR, Rowland LM, Mullins P, et al. 1H-MRS at 4 tesla in minimally treated early schizophrenia. *Mol Psychiatry.* 2010;15(6):629-636.

168. Egerton A, Broberg BV, Van Haren N, et al. Response to initial antipsychotic treatment in first episode psychosis is related to anterior cingulate glutamate levels: a multicentre (1)H-MRS study (OPTiMiSE). *Mol Psychiatry.* 2018;23(11):2145-2155.

169. Legind CS, Broberg BV, Mandl RCW, et al. Heritability of cerebral glutamate levels and their association with schizophrenia spectrum disorders: a (1)[H]-spectroscopy twin study. *Neuropsychopharmacology.* 2019;44(3):581-589.

170. Taylor R, Osuch EA, Schaefer B, et al. Neurometabolic abnormalities in schizophrenia and depression observed with magnetic resonance spectroscopy at 7 T. *BJPsych Open.* 2017;3(1):6-11.

171. Theberge J, Al-Semaan Y, Williamson PC, et al. Glutamate and glutamine in the anterior cingulate and thalamus of medicated patients with chronic schizophrenia and healthy comparison subjects measured with 4.0-T proton MRS. *Am J Psychiatry.* 2003;160(12):2231-2233.

172. Yang Z, Zhu Y, Song Z, et al. Comparison of the density of gamma-aminobutyric acid in the ventromedial prefrontal cortex of patients with first-episode psychosis and healthy controls. *Shanghai Arch Psychiatry.* 2015;27(6):341-347.

173. Moher D, Liberati A, Tetzlaff J, Altman DG, Group P. Preferred reporting items for systematic reviews and meta-analyses: the PRISMA Statement. *Open Med.* 2009;3(3):e123-130.
